# Supplementary material for: TElehealth in CHronic disease: mixed-methods study to develop the TECH conceptual model for intervention design and evaluation
Source: BMJ Open. 2015 Feb 6;5(2):e006448. doi: 10.1136/bmjopen-2014-006448 (PMC4322202; doi:10.1136/bmjopen-2014-006448)

BMJ Open

Telehealth to support patients with chronic conditions: development and use of a conceptual model to guide intervention design and evaluation

Journal:	*BMJ Open*
Manuscript ID:	bmjopen-2014-006448
Article Type:	Research
Date Submitted by the Author:	22-Aug-2014
Complete List of Authors:	Salisbury, Chris; University of Bristol, Centre for Academic Primary Care<br>Thomas, Clare; University of Bristol, Centre for Academic Primary Care<br>O'Cathain, Alicia; The University of Sheffield, School of Health and Related Research (ScHARR)<br>Rogers, Anne; University of Southampton, Faculty of Health Sciences<br>Pope, Catherine; University of Southampton, Health Sciences<br>Yardley, Lucy; University of Southampton, Academic Unit of Psychology<br>Hollinghurst, Sandra; University of Bristol, Centre for Academic Primary Care<br>Fahey, Tom; Royal College of Surgeons in Ireland, Department of General Practice<br>Lewis, Glyn; UCL Psychiatric Epidemiology, Mental Health Sciences Unit<br>Large, Shirley; NHS Direct,<br>Edwards, Louisa; University of Bristol, Centre for Academic Primary Care<br>Rowsell, Alison; University of Southampton, Health Sciences<br>Segar, Julia; University of Manchester, Centre for Primary Care<br>Brownsell, Simon; University of Sheffield, School of Health and Related Research (ScHARR)<br>Montgomery, Alan; University of Nottingham,
**Primary Subject Heading**:	General practice / Family practice
Secondary Subject Heading:	Health informatics, Health services research, Mental health, Cardiovascular medicine
Keywords:	Telemedicine < BIOTECHNOLOGY & BIOINFORMATICS, PRIMARY CARE, Hypertension < CARDIOLOGY, Organisation of health services < HEALTH SERVICES ADMINISTRATION & MANAGEMENT, Depression & mood disorders < PSYCHIATRY

SCHOLARONE™
Manuscripts

Telehealth to support patients with chronic conditions: development and use of a conceptual model to guide intervention design and evaluation

Authors names, degrees, affiliations:

Professor Chris Salisbury, MD FRCGP [1]
Dr Clare Thomas, PhD[1]
Professor Alicia O'Cathain, PhD [2]
Professor Anne Rogers, PhD [3]
Professor Catherine Pope, PhD [3]
Professor Lucy Yardley, PhD [4]
Dr Sandra Hollinghurst, PhD[1]
Professor Tom Fahey, MD [5]
Professor Glyn Lewis, PhD FRCPsych [6]
Dr Shirley Large, PhD [7]
Dr Louisa Edwards PhD [1]
Ms Alison Rowsell, MPhil [4]
Dr Julia Segar, PhD [8]
Dr Simon Brownsell, PhD [2]
Professor Alan A Montgomery, PhD [9]

[1] **University of Bristol**
Centre for Academic Primary Care, School of Social and Community Medicine
Canynge Hall, 39 Whatley Road, Bristol, BS8 2PS

[2] **University of Sheffield**
Medical Care Research Unit, School of Health and Related Research (ScHARR)
Regent Court, 30 Regent Street, Sheffield, S1 4DA

[3,4] **University of Southampton**
[3] School of Health Sciences, Building 67, Highfield, Southampton, Hampshire, S017 1BJ
[4] Centre for Applications of Health Psychology, Building 44, Highfield, Southampton SO17 1BJ

[5] **HRB Centre for Primary Care Research, Royal College of Surgeons in Ireland**
Medical School, Department of General Practice, 123 St Stephens Green, Dublin 2, Ireland

[6] **University College London**

For peer review only

Division of Psychiatry, University College London
67-73 Riding House Street, London, W1W 7EJ

[7] **NHS Direct**
Strawberry Fields, Berrywood Business Village, Tollbar Way,
Hedge End, Hampshire, SO30 2UN

[8] **The University of Manchester**
Centre for Primary Care, Institute of Population Health
5th Floor Williamson Building, Oxford Road, Manchester, M13 9PL

[9] **University of Nottingham**
Nottingham Health Science Partners, Nottingham Clinical Trials Unit
C Floor, South Block, Queen's Medical Centre, Nottingham, NG7 2UH

Corresponding author:

Professor Chris Salisbury
Centre for Academic Primary Care, School of Social and Community Medicine, University of Bristol, Canynge Hall, 39 Whatley Road, Bristol, BS8 2PS, UK

Tel: +44 (0)117 331 4530 **Email:** c.salisbury@bristol.ac.uk

Keywords
Telemedicine, Chronic disease, Theoretical models, Cardiovascular diseases, Depression.

Words: 4374

For peer review only

Abstract

Objective: To develop a conceptual model for effective use of telehealth in the management of chronic health conditions, and to use this to develop and evaluate an intervention for people with two exemplar conditions: raised cardiovascular disease risk and depression.

Design: The model was based on several strands of evidence: a meta-review and realist synthesis of quantitative and qualitative evidence on telehealth for chronic conditions; a qualitative study of patients' and health professionals' experience of telehealth; a quantitative survey of patients' interest in using telehealth; a review of existing models of chronic condition management; a review of evidence-based guidelines. Based on these evidence strands, a TECH model was developed and then refined at a stakeholder workshop. Finally, a telehealth intervention ('Healthlines') was designed by incorporating strategies to address each of the model components. The model also provides a framework for evaluation, which is being undertaken through parallel randomized controlled trials in the two exemplar conditions.

Setting: Primary care

Results: The Telehealth in Chronic Disease (TECH) model has four main components: (i) engagement of patients and health professionals (ii) effective chronic disease management (including sub-components of self-management, optimization of treatment, care co-ordination) (iii) partnership between providers (iv) patient, social and health system context. Key intended outcomes are improved health, access to care, patient experience and cost-effective care.

Conclusions: A conceptual model has been developed based on multiple sources of evidence which articulates how, why and under what circumstances telehealth may provide benefits for patients with chronic health conditions. Based on evidence-based components and stakeholders' views, it structures the design and evaluation of telehealth programs which are likely to be acceptable to patients and providers and also cost-effective.

For peer review only - http://bmjopen.bmj.com/site/about/guidelines.xhtml

Article summary

Strengths and limitations of this study

- This paper describes the development and use of an evidence based conceptual model for the effective use of telehealth amongst patients with chronic conditions
- Having a conceptual model provides a framework for intervention development and evaluation
- The model is now being evaluated through parallel randomised controlled trials in two exemplar chronic conditions
- In order to develop a model which is clear, simple and generalisable there is a risk of over-simplification of the multiple mechanisms by which telehealth may have its effects
- The strength of evidence available to justify different components of the conceptual model is variable

For peer review only

For peer review only - http://bmjopen.bmj.com/site/about/guidelines.xhtml

Introduction

The role of telehealth in chronic health conditions

There is international interest in the potential of telehealth to support the management of patients with chronic health conditions. Telehealth refers to the use of electronic and telecommunication technologies to support health care at a distance from the patient. This reflects a recognition that, as the population ages, the needs of the increasing number of people with chronic conditions are likely to overwhelm the capacity of conventional health care services designed around scheduled one-to-one, face-to-face, appointments between patients and doctors. In the United Kingdom (UK) 30% of the population have at least one chronic condition and they account for 70% of total health services expenditure.[1] There is a need to harness the potential of technology to support people to manage themselves in their own homes. This has potential to shift the locus of control so that, through better access to information, people can become experts in their own care. Provision of health care at a distance (for example through telemonitoring) could in theory be more accessible, efficient and responsive than patients or professionals having to travel to face-to-face appointments.

Considerable resources have been committed to implementing different forms of telehealth for chronic conditions. For example, in the United States the Veterans Health Administration introduced a national home telehealth program which had enrolled about 50,000 patients by 2011,[2 3] the Renewing Health Consortium is developing and testing telehealth programme in 9 European countries,[4] while in the UK the Whole System Demonstrator project was established to provide telehealth at scale for patients with conditions such as heart failure or chronic lung disease.[5-7]

Evidence of benefits

Although the potential benefits of telehealth in chronic condition management have been rehearsed for at least 20 years, evidence to support these arguments is limited.[8-10] Systematic reviews have been conducted in specific chronic conditions, along with overviews which have combined findings from a range of conditions, which have concluded that the evidence in favour of telehealth is weak and inconsistent. [8 9 11-16] Evidence of effectiveness is stronger for some conditions (for example, heart failure) than it is for others (for example, diabetes). Some studies report positive findings while others do not, and it has been difficult to identify a pattern in terms of disease, type of technology, or patient characteristics to explain these inconsistencies. There is a lack of evidence about mechanisms of action and about wider impacts of telehealth on utilization of other health care services.[9] There is inconsistent reporting of outcomes, suggesting a lack of clarity about the intended benefits of telehealth and making it difficult to compare studies. Evidence about cost-effectiveness or of successful wide scale implementation is particularly limited.

For peer review only - http://bmjopen.bmj.com/site/about/guidelines.xhtml

For peer review only

The need for a conceptual model

Telehealth is a complex intervention, [10 17] involving a number of interacting components, such as the type of technology, the infrastructure, the human support available, and the capabilities of the patient in relation to the technology. For any individual, telehealth is likely to be only one facet of the health care they receive, so telehealth cannot be understood in isolation from the health care system in which it is provided.

Over the last 15 years there has been increasing awareness of the importance of theory both in the development and evaluation of complex interventions.[18] Theory is needed in order to understand the relationship between context, mechanism of action and intended outcomes, but this has largely been neglected in the field of telehealth.[19-21] While there are well recognised theories in related topics such as behaviour change (e.g. the Theory of Planned Behavior[22], the Behavior Change Wheel[23], Ritterbrand[24]), and why technologies get used (for example, the Technology Acceptance Model[25]), there is no over-arching theory which connects these and other elements (such as co-ordination between service providers) essential to chronic disease management in the context of telehealth.

What is needed is a clear conceptual model for how and why a telehealth intervention for patients with chronic conditions is intended to have specified beneficial effects. Making explicit the theoretical chain of causation by which an intervention is intended to lead to its effects focuses attention on the most important features of the intervention that need to be delivered for it to be effective. A conceptual model also provides a framework for evaluation by identifying the contextual factors, steps in the causal chain and most important outcomes that need to be assessed. To be practically useful, a conceptual model should be sufficiently generalizable to apply to a range of conditions, types of interventions and health care settings.

This paper describes the development of a conceptual model for the role of telehealth in the management of chronic conditions. This was developed to inform the design of an intervention to support people with two exemplar conditions: raised cardiovascular disease risk (due to risk factors such as hypertension, smoking, obesity and hyperlipidaemia) or depression. These exemplars were chosen to represent very different types of condition, which would test the generalizability of the model, but which are both common and where there was existing evidence that some forms of telehealth could be effective.[26 27] By taking into account the views of patients and providers and considerations about cost as well as evidence of effectiveness, the intention was to develop a model for interventions which are likely to be suitable for implementation at wide scale, acceptable to stakeholders, and cost-effective.

For peer review only - http://bmjopen.bmj.com/site/about/guidelines.xhtml

Methods

Evidence review

The model was based on several sources of evidence. The methods and results for each strand of evidence are summarized below, but are described in more detail elsewhere.

1. A **meta-review and realist synthesis** of existing quantitative and qualitative evidence on telehealth for chronic conditions.[16] This consisted of an overview of existing systematic reviews of telehealth interventions. We focused on reviews of chronic conditions generally rather than in relation to specific conditions. We included telephone and internet-based interventions (such as telecoaching, telephone/internet counselling and follow-up) and telemonitoring of symptoms and vital signs, but not telemedicine approaches where technologies are used to share information between healthcare providers. We searched Medline, CINAHL, Embase, AMED, PsycINFO, and the Cochrane Library databases for high quality systematic reviews about telehealth and chronic conditions published in English between January 2005 and March 2010. Two reviewers independently reviewed abstracts and (where relevant) full papers and extracted data onto a standardized form. We supplemented the meta-review with a new systematic review to look in more detail at studies of telehealth interventions focused on telehealth interventions for prevention of cardiovascular disease.[28] In addition we identified and reviewed published qualitative studies of patients' experience of using telehealth interventions. In total, we included 16 systematic reviews (representing 662 quantitative studies) and 29 qualitative studies. We combined these sources of data in a realist synthesis in which we sought to identify mechanisms of action for telehealth in chronic conditions. Realist synthesis is an approach to reviewing research evidence on complex interventions in order to provide an explanatory analysis for how and why they work (or don't work) in particular contexts or settings.[29]
2. A **qualitative study** of the potential role of telehealth in chronic conditions[30]. This involved interviews and observation with patients as well as doctors and nurses providing primary care for patients with chronic conditions, and health information advisors who provided an existing telephone based health coaching and care management service for patients with chronic conditions such as heart failure or diabetes.[31] Semi-structured interviews were conducted with 38 patients and 68 health professionals and observation was undertaken at a call centre providing telehealth. The research took place between April 2010 and March 2011. Thematic analysis of qualitative data was undertaken.
3. A **survey of patients** to assess relationships between patient characteristics, health needs, difficulties with access to health care, attitudes towards and availability of various technologies, and interest in using different types of telehealth.[32] Patients

For peer review only - http://bmjopen.bmj.com/site/about/guidelines.xhtml

with either raised cardiovascular risk (n=872) or depression (n=606) were identified and randomly sampled from 34 general practices in two areas of the UK and invited to complete a questionnaire.

4. A **review of existing models of chronic condition management** (not necessarily involving telehealth) to identify common factors in these models which appeared to be associated with improved care and benefits for patients.
5. A **review of national guidelines** from the US, UK and Europe for our exemplar conditions to identify the main recommendations and priorities for treatment. We cross-referenced these with our meta-review to identify evidence for the effectiveness of telehealth interventions to address these priorities (for example, the use of online programs to deliver cognitive behavioural therapy for depression or the use of home monitoring of blood pressure in patients with hypertension).

Synthesis

We synthesized the findings from our evidence review in two stages. First, it was clear from the meta-review and the qualitative study that engagement with both patients and professionals was key to the success of a telehealth intervention. We therefore used a modified PRECEDE-PROCEED[33] approach to intervention development in which we used the insights from our evidence sources to map the predisposing, enabling and reinforcing factors that determine engagement with telehealth, creating separate maps for patients and health professionals. Through discussion within the research team we listed and grouped themes from the literature reviews, qualitative research and patient survey, cross-referenced to the sources of evidence. Next, commonalities across these three sources of evidence were highlighted and key themes identified.

Second, we developed a draft model for the use of telehealth to support the management of chronic conditions which encapsulated the findings from the evidence review. We labelled this the TECH model (Telehealth for Chronic Diseases). Several different layouts and versions of the model were discussed iteratively in meetings of the research team until we had developed a draft model. Finally, we convened an intensive one day workshop for a wide range of stakeholders (n= 38) including patients, care providers, managers, commissioners of services, independent academics and the research team in which we discussed the findings of our evidence review in order to refine the final model.

Using the model to design an intervention

The research team used the TECH conceptual model to design a telehealth patient management program, known as the Healthlines service. This was designed to be delivered by NHS Direct, which (at the time the intervention was designed) provided health information and advice throughout England based on a network of telephone call centres and an associated website. The intention was to design an intervention that would be likely to be cost-effective by maximizing patient benefit at minimum cost, and which could

For peer review only - http://bmjopen.bmj.com/site/about/guidelines.xhtml

feasibly be rolled out quickly on a national scale if it proved to be effective. For these reasons, the design of the intervention sought to incorporate technologies which were already available and approaches for which there was already some evidence of effectiveness. We avoided cutting-edge technologies that were not already developed or tested and high cost solutions that would be unlikely to be widely available or deliverable to large numbers of patients. In order to maximize population benefit the aim was to focus on the large number of patients at moderate risk of health problems (e.g. patients with hypertension and other cardiovascular risk factors) rather than the small number of patients at high risk (for example, patients who have already had a stroke).

The research team used the patient and professional 'maps' generated through the PRECEDE-PROCEED method to develop strategies to promote engagement with the telehealth intervention by addressing each of the predisposing, enabling and reinforcing factors previously identified.

The model as a framework for evaluation

The Healthlines service is being evaluated within two pragmatic parallel randomized controlled trials. We recruited 43 general practices providing primary health care in three areas of England. Adult patients from these practices with either (a) raised risk of a first cardiovascular event (10 year risk >20%) or (b) depression were recruited to take part and were individually randomized to receive either usual primary care plus extra support from the Healthlines service or usual primary care alone. The protocol for these trials has been published (Trial Registration: Current Controlled Trials: cardiovascular disease risk trial ISRCTN27508731 and Depression trial ISRCTN14172341).[34]

The TECH conceptual model is being used to provide a framework for evaluation by describing the extent to which each element of the model was successfully delivered and the intended outcomes were achieved.

Results

Evidence review

Meta-review, realist synthesis, qualitative study and quantitative patient survey:

Key findings from these studies are summarized in Box 1.

For peer review only - http://bmjopen.bmj.com/site/about/guidelines.xhtml

Box 1: Key findings from the meta-review, qualitative study and patient survey

Meta-review[16 28]

- Some evidence of improvements in clinical outcomes
- Much of the primary research is of poor quality and limited to short term effects
- Evidence about impact on the wider health care system and cost-effectiveness is sparse
- Inconsistent findings about effectiveness and resource utilization, with few clear patterns in terms of types of patient, disease or technology associated with benefits
- Many telehealth interventions for chronic conditions have struggled to engage both patients and health care professionals, with low uptake and high drop-out rates.
- Simple technologies, especially those based on telephone support, have at least as strong an evidence base as more sophisticated technologies such as tele-monitoring
- Telephone support seems to enhance the benefit of web based technology

Realist synthesis

This suggested three key mechanisms by which telehealth worked to improve health outcomes:

- Relationships: good connections between patients, peer groups and/or professionals provide support
- Fit: Acceptability, ease of use, and integration into everyday routines were important to both patients and professionals
- Visibility: Monitoring provides feedback, reinforcement and prompts to change behaviour but can also have negative connotations of surveillance

Qualitative study[30]

- Nurses and doctors working in primary care were ambivalent about the contribution of telehealth to chronic condition management, because of concerns about the lack of evidence of benefit, duplication of their own work and a threat to their role
- There is a need to take account of how new telehealth programs integrate with existing health system structures
- Patients were more likely to trust a telehealth system if it is endorsed by their usual primary care providers
- Patients valued a personal approach based in human interaction

Patient survey[32]

- There was moderately strong interest in telehealth support for chronic conditions across all age groups
- There was greatest interest in telephone and internet based interventions, and minimal interest in social media, particularly amongst older patients with chronic conditions
- There was little relationship between health care need or difficulties in accessing health care and interest in telehealth
- The most important constructs associated with interest in telehealth were confidence in using the technology and perceived advantages and disadvantages of telehealth
- Interest in telehealth was not related to patient socio-demographic variables, after adjusting for modifiable factors such as access to and confidence in using the technology

For peer review only - http://bmjopen.bmj.com/site/about/guidelines.xhtml

For peer review only

Existing models of chronic condition management

We identified a number of existing models for the management of chronic conditions, but the dominant approach is the Chronic Care Model (CCM).[35] A number of studies have suggested that programmes based on the CCM can improve health outcomes for a range of chronic conditions, although it is uncertain which components of the model are most important or whether all are necessary.[36-38] The CCM includes elements which relate to national aspects of the health care system and does not in itself provide a model for the design of telehealth interventions. Between 2003 and 2007, the Veterans Administration introduced a national home telehealth program, Care Coordination/Home Telehealth (CCHT),[2] which was strongly influenced by the CCM but applied the concepts more specifically to telehealth applications in a United States context.

Review of national guidelines

In order to apply a conceptual model to a specific condition the key health problems and care needs must be identified. For raised cardiovascular disease risk, our review of international guidelines suggested that these were the modifiable risk factors of hypertension, smoking, obesity, raised cholesterol, and lack of exercise. Evidence based priorities for intervention included optimizing drug treatment in order to achieve blood pressure targets, ensuring medication adherence, providing nicotine replacement therapy for smokers along with behavioural support, providing advice about diet and exercise and referral to weight management programs for obesity, and ensuring that statins were prescribed and taken.

For depression, the priorities for intervention included offering psychological therapies such as cognitive behavioural therapy and/or anti-depressant drug treatment with intensity of treatment tailored in relation to need, having relapse prevention strategies, ensuring medication adherence, offering peer support, avoiding alcohol misuse, encouraging exercise, and assessing suicidal risk.

Synthesis and developing the model

Figure 1 shows the final TECH model. This proposes that it is essential for telehealth interventions to incorporate four key concepts in order to support chronic conditions.

1. Engagement of patients and primary care providers

The literature meta-review highlighted that many telehealth interventions have been unsuccessful because of low uptake by patients and high rates of drop-out. Both our qualitative research and the patient survey illustrated the range of factors that act as motivators or barriers to patients using telehealth. These are summarized in Box 2 based on our PRECEDE-PROCEED map of predisposing, enabling and reinforcing factors for patients.

For peer review only - http://bmjopen.bmj.com/site/about/guidelines.xhtml

Box 2: Predisposing, enabling and reinforcing factors to the use of telehealth by patients

Pre-disposing

- Attraction of having support for health problems on demand, having more time, getting greater support
- Patients having a clear understanding of why they have been included
- Confidence in ability to use the technology
- Being reassured about privacy and confidentiality

Enabling

- Good access to fast reliable internet connection
- Technology which is simple and inexpensive, not complicated to use

Reinforcing

- Benefits of having regular review
- Importance of self-monitoring which promotes continued engagement
- Encouraging patient activation and involvement rather than passive reminders

With regard to helth care professionals, our qualitative research indicated that many were unenthusiastic and, in some cases, resistant towards telehealth interventions. Our PRECEDE-PROCEED map for professionals identified several factors that were likely to influence engagement in telehealth. These included the belief that medicine should be evidence-based, and scepticism about the evidence for telehealth (pre-disposing factor), concerns about duplication of care (pre-disposing), the need for technology to be simple and reliable (enabling), and the importance of clarity of roles for conventional and telehealth providers and good communication between them (reinforcing).

2. Effective chronic disease management:

Our evidence synthesis and review of existing models of chronic condition management suggested that strategies that contribute to effective care and which could be delivered via telehealth can be summarized under three headings: promoting self-management, optimizing treatment, and care coordination. The various strategies that comprise each of these headings are shown in Box 3, along with citations for specific studies or reviews that provide evidence of effectiveness for each element (not necessarily in the field of telehealth).

For peer review only - http://bmjopen.bmj.com/site/about/guidelines.xhtml

Box 3: Components of effective chronic condition management

Promoting self-management

- Behaviour change techniques, e.g. stimulus control, problem solving, cognitive restructuring, goal setting[39 40]
- Self-monitoring [27 41 42]
- Provide patient information [43 44]
- Promote self-efficacy [45-47]
- Shared decision making [44]
- Motivational interviewing [39 40]
- Personal support from health professionals [48 49]

Treatment optimisation

- Risk stratification with case management for complex patients [50 51]
- Treatment intensification [49 50 52 53]
- Use of evidence-based guidelines and protocols [49 53]
- Regular review [44 50 52]
- Promote medication adherence [40 44]
- Share treatment recommendations with patients [54]

Care co-ordination

- Interventions that included multiple reinforcing components [40 44 48]
- Shared records, information and treatment recommendations between patients, primary care and the telehealth provider [2 47]
- Communication (remote and face-to-face) between the telehealth provider and primary care [2]
- Regular monitoring of system performance [38 55]
- Seek to support rather than duplicate primary care [56]

3. Partnership

Our qualitative research highlighted that a telehealth intervention is just one aspect of the health care provided to a patient with a chronic condition. These patients are likely to continue to get the majority of their care from their family practitioner, with whom they may have had a long-term relationship, and whom they will continue to consult for reasons apart from their chronic condition. In addition, many patients with chronic conditions are likely to be receiving help from hospital specialists and other health and social care agencies.

However, our evidence review suggested that many previous telehealth interventions appear to have failed because they were designed in isolation from the rest of the health care system, leading to duplication of effort, lack of co-ordination between providers, inefficiency and confusion for patients. This is likely to reinforce the resistance expressed by

For peer review only - http://bmjopen.bmj.com/site/about/guidelines.xhtml

other health care providers. Our qualitative research showed that these other providers may perceive the telehealth intervention to be an unnecessary interference in their area of responsibility, possibly representing a threat to their future role.

Therefore, it is important for a model for telehealth interventions to emphasize that telehealth should be delivered in partnership, identifying the role that telehealth can play to support rather than compete with patients' main primary health care providers.

4. Context: characteristics of patients and wider social and health system

The patient survey and the literature review both indicated that characteristics of patients are likely to have an impact on how telehealth affects outcomes. These include socio-demographic characteristics, particularly age, the nature of their chronic condition, and the severity of their condition. The design of a telehealth intervention must also take account of the wider social and health system context.[57 58] For example a program designed to work within a health system context with a strong primary care foundation may need different features from one designed for a system in which patients consult different hospital specialists for each of their chronic conditions. Similarly, a system which assumes that patients have access to fast and reliable internet connections will not work where this does not apply. Finally, different funding models for health care create different financial incentives for providers and patients which may have a major influence over how telehealth systems are implemented.

Specifying outcomes

The TECH model depicted in Figure 1 seeks to capture the four components of the model in a way that is conceptually clear, simple and generalizable. It also proposes the improved outcomes that telehealth interventions are intended to deliver for patients with chronic conditions. These are improved health outcomes, access to care and patient experience, care provided in a way which is cost-effective. One criticism of earlier research on telehealth interventions has been the lack of consistency in reporting outcomes,[8] and this model provides a framework for the outcomes that should be assessed in future evaluations, as well as potential mediators in order to gain understanding of the mechanism of action.

Using the model to design a telehealth intervention

We used the conceptual model to design telehealth intervention programs to support the management of patients with (a) raised cardiovascular risk or (b) depression. We used the same model to design interventions which were similar in concept but different in terms of detailed content to address each of the priority health and care needs for these two groups of patients, based on our review of national guidelines.

Table 1 provides examples of how we devised strategies to be delivered within the Healthlines service to populate the conceptual model for the intervention to be used for cardiovascular risk. Appendix 1 provides an expanded and more comprehensive list of the

For peer review only - http://bmjopen.bmj.com/site/about/guidelines.xhtml

strategies we used for both raised cardiovascular risk and depression, and the Healthlines service has also been described in detail elsewhere.[34]

Table 1 Use of the TECH model to design the Healthlines telehealth intervention for patients with raised cardiovascular risk

MODEL ELEMENT	STRATEGIES INCLUDED IN INTERVENTION
**Engagement**	
Patient	Healthlines advisors provide simple welcome pack and technical support to overcome lack of confidence in technology
	Encourage sense of personal care through seeking to maximize continuity of care from one named Healthlines advisor
Health professional	All communications seek to reinforce message that the Healthlines service is supporting and delivered alongside primary care
	Messages to primary care emphasize evidence based nature of interventions and guidance
**Promoting self-management**	
Behaviour change techniques	Healthlines cardiovascular intervention adapted from the Duke self-management package,[59] which uses scripts for advisors based on psychological principles of behaviour change. Intervention is tailored to patient's needs and goals.
Self-monitoring & feedback	Provide patients with free BP monitors and web-site to log readings which gives immediate feedback and graphical display about whether BP is above or below target (Appendices 2 and 3)
Provide patient information	Healthlines advisor works with patients to identify goals and then emails them links to further resources available on the internet, which have been quality assessed (e.g. diet advice, risk calculators, videos, patient forums)
**Treatment optimization**	
Risk stratification	Calculate cardiovascular risk. Level of intervention guided by level of risk factor with escalation to GP for patients at high risk
Treatment intensification	Monthly review of BP using online log of BP readings, protocol driven advice to GP to intensify treatment each month if targets not met
Promote medication adherence	Monthly review of medication adherence, scripts use evidence based strategies to improve adherence, GPs advised by email if patients appeared to be non-adherent
**Care co-ordination**	
Shared records	All treatment recommendations shared with both primary care provider and patient. A summary of recent BP records from patient web-portal is sent to GP when treatment change is recommended.
Regular monitoring of system performance	Reporting module which allows monitoring of management program (e.g. of number of patients who have been telephoned, number actively self-monitoring BP)
**Partnership**	
	All communications are shared between Healthlines, GP and patient. Communication is two way: GPs can contact Healthlines e.g. to change a patient's BP target
	GPs and service managers involved in designing the Healthlines intervention
**Context**	
	Not all patients in UK have access to reliable internet connections. It is important to describe the characteristics of patients who take part, for evaluation.

For peer review only - http://bmjopen.bmj.com/site/about/guidelines.xhtml

Use of the TECH model for evaluation

The TECH model proposes four main outcomes resulting from telehealth interventions for chronic disease, the first of which is improved health outcomes. For the cardiovascular trial the primary outcome is cardiovascular risk status 12 months following randomization. For depression, the primary outcome is a clinically significant improvement in depression. Secondary outcomes for both trials include health related quality of life, measures of access to health care and patient satisfaction with care. An economic analysis will assess cost-effectiveness over the 12 months of the trial, and for the cardiovascular risk trial will also model the long-term costs and benefits of the intervention after taking into account the predicted number of strokes and heart attacks over the next 10 years.[34]

Alongside the randomized controlled trial, a process evaluation will explore the extent to which the intervention was delivered as intended, and whether it led to the expected changes at each step of causal chain hypothesized by the conceptual model. Therefore it will be important to assess patient characteristics and health service context, patient and primary care engagement, patient self-management, treatment optimisation, care co-ordination and partnership with other health care providers, as well as the primary and secondary outcomes described above. These will be assessed using validated measures where possible. Qualitative research through interviews with patients, primary care health professionals and Healthlines advisors will be conducted to understand in greater detail how the service was delivered, barriers and facilitators to implementation, and how and why the intervention did or did not appear to be effective from the perspectives of those delivering and receiving it.

Discussion

Principal findings

This article describes the development of the TECH conceptual model for the effective use of telehealth amongst patients with chronic conditions and illustrates how it has been used to design and evaluate telehealth interventions for patients at either raised risk of cardiovascular disease or depression. If these evaluations in different chronic conditions are positive, this will provide support for the model about how this type of telehealth intervention works, suggesting it can then be applied to other chronic conditions.

Alternatively, if the intervention is unsuccessful, it will be possible to assess each of the processes in the causal chain in order to determine whether the intervention was not delivered as intended, or whether the assumed causal relationships were incorrect. For example, the model posits that one way in which telehealth works is by allowing people to monitor their own health, which will lead to changes in their behaviour, which will have a positive impact on their health. Having a model highlights the need to assess the extent to which participants actually did self-monitoring as intended, whether this was associated

For peer review only - http://bmjopen.bmj.com/site/about/guidelines.xhtml

with behaviour change, and whether this led to improved health outcomes. This kind of approach provides a framework for correction and adaptation of an intervention through understanding which intervention components are more or less effective at impacting proximal outcomes in the causal chain.[60]

Strengths and limitations

The strength of this research is that we have used diverse sources of evidence to develop a conceptual model which creates a framework for intervention development and evaluation. Each of the components of the model can be justified from our own research and evidence from previous literature.

Recognizing that the simplest models have the greatest utility, we sought to provide a simple graphical depiction of the causal chain in a successful telehealth intervention. However, we recognize that the model diagram over-simplifies the multiple potential mechanisms by which a telehealth intervention may have its effect. There are likely to be associations and interactions between different elements of the model, and both recognized and unrecognized confounding factors. However, to indicate all of these potential relationships in the model would, in our view, reduce its usefulness in providing a framework.

A further limitation is that the strength of underlying evidence to support each of the components of the model is variable. For example, evidence of the benefit of patient self-monitoring is strong for some chronic conditions, but not all, and although providing patient information and shared decision making are viewed as important aspects of chronic condition management in the Chronic Care Model and other similar models, the evidence that these strategies lead to improved patient outcomes is limited. Nevertheless, we have sought to include components in the model where the overall weight of evidence supports their value.

Relationship to previous studies

Several previous authors have argued for the importance of theory in designing telehealth interventions, from a range of perspectives,[19 55 61]and there are also existing frameworks for the assessment (rather than the design) of telehealth for chronic conditions, such as the Model for Assessment of Telemedicine (MAST).[62] The intervention which is most relevant to our study and well described in terms of its underlying theoretical basis is the Comprehensive Health Enhancement Support System (CHESS), an umbrella term for several e-health programs combining information, adherence strategies, decision-making tools and support services.[60 63] Like the Healthlines intervention described here, CHESS was developed by combining several intervention features each of which had some theoretical justification. However, CHESS was developed without any clear theory about how the program features related to each other,[60] and the TECH model underpinning the Healthlines intervention is intended to address this limitation.

For peer review only - http://bmjopen.bmj.com/site/about/guidelines.xhtml

Greenhalgh et al have taken a more radical stance and argued against the quasi-experimental approach advocated by previous authors in favour of in-depth case studies, viewing program evaluation not as experimentation but as social practice.[64] They claim that there is a need to recognize the complex political dynamics and language games practiced by different stakeholders and to question rationalist assumptions about 'what works'.[64] We recognize the importance of these political considerations in how telehealth programs are implemented and evaluated, and in how the findings from such evaluations are sometimes interpreted to fulfil a prior agenda. However, this does not undermine the need to develop interventions based on a understanding of how and in what ways telehealth programs might be effective; indeed a clear theoretical basis for interventions and clarity about intended outcomes might provide the most robust defence against selective use of findings and may allow a more nuanced understanding about why interventions are more or less effective in different contexts.

Implications for clinicians and policy-makers

This paper describes a clear conceptual model, based on several sources of evidence, which helps to articulate the theoretical basis for how, why and under what circumstances telehealth could provide specified benefits for patients with chronic health conditions. Because it is based on evidence-based components and the views of stakeholders, the TECH model provides the basis for the design of telehealth interventions which are likely to be effective, cost-effective and acceptable to patients and health care providers. Importantly, it also provides a framework for evaluation.

For peer review only - http://bmjopen.bmj.com/site/about/guidelines.xhtml

Funding

This article presents independent research funded by the National Institute for Health Research (NIHR) under Healthlines program (RP-PG-0108-10011). The views expressed in this publication are those of the author(s) and not necessarily those of the NHS, the NIHR, or the Department of Health.

Competing interests

The authors have no competing interests

Contributors

CS, A O'C, AR, CP, LY, SH, TF, GL, SL, SB and AM had the idea, developed the protocol, obtained funding and supervised the research. CT managed the research programme. AR, CP and AO'C conducted the meta-review and evidence synthesis. AR and JS conducted the qualitative study. LE and AM conducted the patient survey. All authors contributed to model development. CS drafted the paper which was critically reviewed by all authors. CS is guarantor.

Acknowledgements

We would like to thank the members of the Program Steering Committee, chaired by Professor Brian McKinstry, patient representatives, members of the program advisory group and other stakeholders who contributed to the different components of research which led to the model described in this paper. We would also like to thank Prof Hayden Bosworth for allowing us to adapt the Duke self-management system and NHS Direct for implementing the intervention which is the basis for the trial.

Data Sharing Statement: No additional data available

Appendices

Appendix 1: Use of the model to design the Healthlines telehealth intervention for patients at raised cardiovascular risk or depression

Appendix 2 Web-portal for patients in cardiovascular intervention

Appendix 3 Blood pressure self-monitoring system

For peer review only - http://bmjopen.bmj.com/site/about/guidelines.xhtml

References

1. DH/Long Term Conditions. Long Term Conditions Compendium of Information 2012. https://www.gov.uk/government/uploads/system/uploads/attachment_data/file/216528/dh_134486.pdf.

2. Darkins A, Ryan P, Kobb R, et al. Care Coordination/Home Telehealth: the systematic implementation of health informatics, home telehealth, and disease management to support the care of veteran patients with chronic conditions. Telemedicine and e-Health 2008:1118-26

3. Cruickshank J. Telehealth: what can the NHS learn from experience at the US Veterans Health Administration? 2012. http://www.2020health.org/dms/2020health/downloads/reports/Telehealth-VA/Telehealth%20VA.pdf.

4. Renewing Health: regions of Europa working together for health. http://www.renewinghealth.eu/en/home.

5. Cartwright M, Hirani SP, Rixon L, et al. Effect of telehealth on quality of life and psychological outcomes over 12 months (Whole Systems Demonstrator telehealth questionnaire study): nested study of patient reported outcomes in a pragmatic, cluster randomised controlled trial. BMJ 2013;**346**

6. Henderson C, Knapp M, Fernandez J-L, et al. Cost effectiveness of telehealth for patients with long term conditions (Whole Systems Demonstrator telehealth questionnaire study): nested economic evaluation in a pragmatic, cluster randomised controlled trial. BMJ 2013;**346**:f1035

7. Bower P, Kennedy A, Reeves D, et al. A cluster randomised controlled trial of the clinical and cost-effectiveness of a 'whole systems' model of self-management support for the management of long- term conditions in primary care: trial protocol. Implementation Science 2012;**7**(1):7

8. Wootton R. Twenty years of telemedicine in chronic disease management--an evidence synthesis. J. Telemed. Telecare 2012;**18**(4):211-20

9. Black AD, Car J, Pagliari C, et al. The impact of eHealth on the quality and safety of health care: a systematic overview. . PLoS Medicine / Public Library of Science 2011;**8**(1):e1000387

10. Car J, Huckvale K, Hermens H. Telehealth for long term conditions. BMJ 2012;**344** doi: 10.1136/bmj.e4201.

11. Murray E, Burns J, See TS, Lai R, Nazareth I. Interactive Health Communication Applications for people with chronic disease. Cochrane Database of Systematic Reviews 2005(4):CD004274

For peer review only - http://bmjopen.bmj.com/site/about/guidelines.xhtml

12. Barlow J, Singh D, Bayer S, Curry R. A systematic review of the benefits of home telecare for frail elderly people and those with long-term conditions. J Telemed.Telecare 2007;**13**(4):172-79

13. Pare G, Jaana M, Sicotte C. Systematic review of home telemonitoring for chronic diseases: the evidence base. J. Am. Med. Inform. Assoc. 2007;**14**(3):269-77

14. Deshpande A, Khoja S, McKibbon A, Jadad AR. Real-time (synchronous) telehealth in primary care: systematic review of systematic reviews. Technology Report No 100. Ottawa: Canadian Agency for Drugs and Technologies in Health, 2008.

15. Polisena J, Coyle D, Coyle K, McGill S. Home telehealth for chronic disease management: a systematic review and an analysis of economic evaluations. Int. J. Technol. Assess. Health Care 2009;**25**(3):339-49

16. Pope C, Rowsell A, O'Cathain A, Brownsell S. For want of evidence: a meta-review of home-based telehealth for the management of long-term conditions. 2011. http://www.bristol.ac.uk/healthlines/documents/popeetal.pdf.

17. Lilford RJ, Foster J, Pringle M. Evaluating eHealth: How to Make Evaluation More Methodologically Robust. PLoS Med 2009;**6**(11):e1000186

18. Campbell NC, Murray E, Darbyshire J, et al. Designing and evaluating complex interventions to improve health care. BMJ 2007;**334**(7591):455-59

19. Kaplan B. Evaluating informatics applications--some alternative approaches: theory, social interactionism, and call for methodological pluralism. Int J Med Inform 2001;**64**(1):39-56

20. Mackert M. Expanding the theoretical foundations of telemedicine. J. Telemed. Telecare 2006;**12**(1):49-50

21. Gammon D, Johannessen LK, Sorensen T, Wynn R, Whitten P. An overview and analysis of theories employed in telemedicine studies. A field in search of an identity. Methods Inf. Med. 2008;**47**(3):260-9

22. Ajzen I. The theory of planned behavior. Organ. Behav. Hum. Decis. Process. 1991;**50**(2):179-211

23. Michie S, van Stralen M, West R. The behaviour change wheel: A new method for characterising and designing behaviour change interventions. Implementation Science 2011;**6**(1):42

24. Ritterband LM, Thorndike FP, Cox DJ, Kovatchev BP, Gonder-Frederick LA. A Behavior Change Model for Internet Interventions. nn. Behav. Med. 2009;**38**(1):18-27

25. Davis FD. Perceived usefulness, perceived ease of use, and user acceptance of information technology. MIS Q. 1989;**13**(3):319-40.

For peer review only

For peer review only - http://bmjopen.bmj.com/site/about/guidelines.xhtml

26. Dellifraine JL, Dansky KH. Home-based telehealth: a review and meta-analysis. J Telemed.Telecare. 2008;**14**(2):62-66

27. Pare G, Moqadem K, Pineau G, St-Hilaire C. Clinical effects of home telemonitoring in the context of diabetes, asthma, heart failure and hypertension: a systematic review. Journal of medical Internet research 2010;**12**(2):e21

28. Merriel SWD, Andrews V, Salisbury C. Telehealth interventions for primary prevention of cardiovascular disease: A systematic review and meta-analysis. Prev. Med. 2014;**64C**(0)

29. Pawson R, Greenhalgh T, Harvey G, Walshe K. Realist synthesis: an introduction: ESRC Research Methods Programme, University of Manchester, 2004.

30. Segar J, Rogers A, Salisbury C, Thomas C. Roles and identities in transition: boundaries of work and inter-professional relationships at the interface between telehealth and primary care. Health & Social Care in the Community 2013;**21**(6):606-13

31. Steventon A, Tunkel S, Blunt I, Bardsley M. Effect of telephone health coaching (Birmingham OwnHealth) on hospital use and associated costs: cohort study with matched controls. BMJ 2013;**347:**f4585

32. Edwards L, Thomas C, Gregory A, et al. Are people with chronic diseases interested in using telehealth? J. Med. Internet Res. 2014; 16(5): e123

33. Green L, Kreuter M. *Health program planning: An educational and ecological approach.* . 4th ed. New York, NY: McGrawhill, 2005.

34. Thomas CL, Man MS, O'Cathain A, et al. Effectiveness and cost-effectiveness of a telehealth intervention to support the management of long-term conditions: study protocol for two linked randomized controlled trials. Trials 2014;**15**:36

35. Wagner EH, Austin BT, Von KM. Improving outcomes in chronic illness. Manag.Care Q. 1996;**4**(2):12-25

36. Singh D. Transforming Chronic Care: Evidence about improving care for people with long-term conditions: University of Birmingham, 2005.

37. Bodenheimer T. Interventions to improve chronic illness care: evaluating their effectiveness. Disease management : DM 2003;**6**(2):63-71

38. Ham C. The ten characteristics of the high-performing chronic care system. Health Economics, Policy and Law 2010;**5**(01):71-90

39. Greaves CJ, Sheppard KE, Abraham C, et al. Systematic review of reviews of intervention components associated with increased effectiveness in dietary and physical activity interventions. BMC Public Health 2011;**11**:119

40. Katon WJ, Lin EH, Von KM, et al. Collaborative care for patients with depression and chronic illnesses. N.Engl.J.Med. 2010;**363**(27):2611-20

For peer review only - http://bmjopen.bmj.com/site/about/guidelines.xhtml

41. AbuDagga A, Resnick HE, Alwan M. Impact of blood pressure telemonitoring on hypertension outcomes: a literature review. Telemedicine Journal & E-Health 2010;**16**(7):830-38

42. Agarwal R, Bills JE, Hecht TJ, et al. Role of home blood pressure monitoring in overcoming therapeutic inertia and improving hypertension control: a systematic review and meta-analysis. Hypertension 2011;**57**(1):29-38

43. Roumie CL, Elasy TA, Greevy R, et al. Improving blood pressure control through provider education, provider alerts, and patient education: a cluster randomized trial. Ann. Intern. Med. 2006;**145**(3):165-75

44. NICE. Medicines adherence: involving patients in decisions about prescribed medicines and supporting adherence. CG76. 2011. http://www.nice.org.uk/CG76.

45. Lorig KR, Ritter PL, Laurent DD, Plant K. Internet-based chronic disease self-management: a randomized trial. Med. Care 2006;**44**(11):964-71

46. Lorig KR, Holman H. Self-management education: history, definition, outcomes, and mechanisms. Ann Behav.Med 2003;**26**(1):1-7

47. Solomon M, Wagner SL, Goes J. Effects of a Web-based intervention for adults with chronic conditions on patient activation: online randomized controlled trial. Journal of medical Internet research 2012;**14**(1):e32

48. Bosworth HB, Olsen MK, Grubber JM, et al. Two Self-management Interventions to Improve Hypertension Control: a randomized trial. Ann. Intern. Med. 2009;**151**(10):687-95

49. Green BB, Cook AJ, Ralston JD, et al. Effectiveness of home blood pressure monitoring, Web communication, and pharmacist care on hypertension control: a randomized controlled trial. JAMA 2008;**299**(24):2857-67

50. NICE. Hypertension: Clinical management of primary hypertension in adults. CG127 2011. www.nice.org.uk/guidance/CG127

51. Shea S, Weinstock RS, Starren J, et al. A randomized trial comparing telemedicine case management with usual care in older, ethnically diverse, medically underserved patients with diabetes mellitus. J. Am. Med. Inform. Assoc. 2006;**13**(1):40-51.

52. Glynn LG, Murphy AW, Smith SM, Schroeder K, Fahey T. Interventions used to improve control of blood pressure in patients with hypertension. Cochrane Database of Systematic Reviews 2010(3):CD005182

53. NICE. Depression in adults. The treatment and management of depression in adults. 2009. http://www.nice.org.uk/CG90.

54. McManus RJ, Mant J, Bray EP, et al. Telemonitoring and self-management in the control of hypertension (TASMINH2): a randomised controlled trial. Lancet 2010;**376**(9736):163-72

For peer review only

55. Catwell L, Sheikh A. Evaluating eHealth Interventions: The Need for Continuous Systemic Evaluation. PLoS Med 2009;**6**(8):e1000126.

56. Bodenheimer T. The future of primary care: transforming practice. N. Engl. J. Med. 2008;**359**(20):2086, 89 .

57. McLean S, Protti D, Sheikh A. Telehealthcare for long term conditions. BMJ 2011;**342** doi: 10.1136/bmj.d120.

58. May CR, Finch TL, Cornford J, et al. Integrating telecare for chronic disease management in the community: what needs to be done? BMC health services research 2011;**11**:131

59. Bosworth HB, Olsen MK, Dudley T, et al. Patient education and provider decision support to control blood pressure in primary care: A cluster randomized trial. Am. Heart J. 2009;**157**(3):450-56 .

60. Pingree S, Hawkins R, Baker T, duBenske L, Roberts LJ, Gustafson DH. The Value of Theory for Enhancing and Understanding e-Health Interventions. Am. J. Prev. Med. 2010;**38**(1):103-09

61. Baker LC, Macaulay DS, Sorg RA, Diener MD, Johnson SJ, Birnbaum HG. Effects of Care Management and Telehealth: A Longitudinal Analysis Using Medicare Data. J. Am. Geriatr. Soc. 2013;**61**(9):1560-67

62. Kidholm K, Ekeland AG, Jensen LK, et al. A Model for Assessment of Telehealth Applications: MAST. Int. J. Technol. Assess. Health Care 2012;**28**(01):44-51

63. Gustafson D, Wise M, Bhattacharya A, et al. The effects of combining Web-based eHealth with telephone nurse case management for pediatric asthma control: a randomized controlled trial. Journal of medical Internet research 2012;**14**(4):e101

64. Greenhalgh T, Russell J, Ashcroft RE, Parsons W. Why national eHealth programs need dead philosophers: Wittgensteinian reflections on policymakers' reluctance to learn from history. Milbank Q. 2011;**89**(4):533-63

For peer review only

For peer review only - http://bmjopen.bmj.com/site/about/guidelines.xhtml

Figure 1. TECH Model for telehealth to support patients with chronic conditions

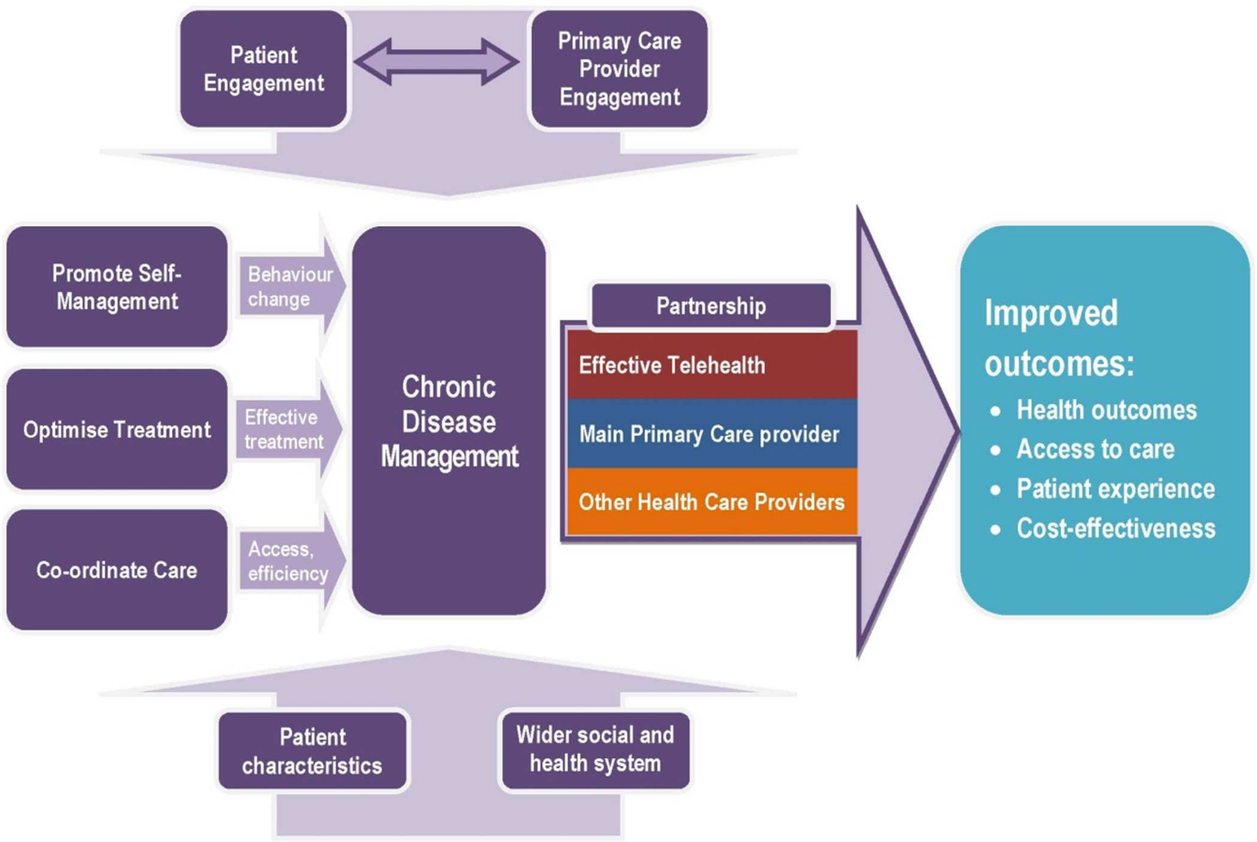

1

For peer review only - http://bmjopen.bmj.com/site/about/guidelines.xhtml

Appendix 1: Use of the model to design the Healthlines telehealth intervention for patients at raised cardiovascular risk or depression

MODEL ELEMENT	STRATEGIES INCLUDED IN INTERVENTION
**Engagement**	
Patient	Provide a 'Welcome Pack'. Emphasize that support with technology will be provided
	Healthlines advisors provide technical support e.g. with getting logged in to websites
	Promote the advantages to patients of using Healthlines, based on perceived advantages identified in qualitative research and other literature, and address perceived disadvantages
	Encourage sense of personal care through seeking to maximize continuity of care from named Healthlines advisor
	Regular positive reinforcement through monthly telephone calls from Healthlines advisor
	Encourage sense of partnership between patient, Healthlines and GP through frequent communication
Health professional	All communications seek to reinforce the message that the Healthlines service is supporting and delivered alongside primary care
	Regular communication with primary care
	Messages to primary care continually emphasize evidence based nature of interventions and guidance
**Promoting self-management**	
Behaviour change techniques	Healthlines cardiovascular intervention adapted from the Duke self-management package, which uses scripts for advisors based on psychological principles of behaviour change. Depression intervention encounters support use of the Living Life to the Full cognitive behaviour course, with additional modules relating to alcohol, exercise, relapse prevention. In both cases, intervention is tailored to patient's needs and goals.
Self-monitoring	CVD risk: Provide patients with free BP monitors and web-site to log readings<br>Depression: Patients using online Living Life to the Full regularly monitor their progress with self-assessment modules including score on PHQ9 questionnaire.
Feedback	CVD risk: BP website gives immediate feedback and graphical display about whether BP is above or below target and next actions
Provide patient	Healthlines advisor works with patients to identify goals and then emails

For peer review only

information	them links to further resources available on the internet which have been quality assessed (e.g. diet advice, risk calculators, videos, patient forums)
Promote self-efficacy	Using motivational interviewing approach, identify motivating factors, encourage action plans and goal setting
Motivational interviewing	All Healthlines advisors undertake motivational interviewing training
Shared decision making	Make targets (e.g. for BP) explicit to patients, provide information about advantages and disadvantages of treatments, encourage patients to discuss options with GP, share letters to GPs with patients
Personal support from health professionals	As far as possible, provide continuity of care from one named Healthlines advisor rather than an anonymous 'call-centre' approach
Peer support	Patients in depression intervention are offered option to access Big White Wall, an online forum for patients with depression
**Treatment optimization**	
Risk stratification	CVD: Calculate cardiovascular risk using QRISK. Level of intervention guided by level of risk factor with escalation to GP for patients at high risk<br>Depression: Assessment using PHQ9 and advice about treatment in relation to severity. PHQ9 also used to assess suicidal risk with use of a protocol for escalation and more detailed risk assessment for patients at significant risk
Treatment intensification	CVD: Monthly review of BP using online log of BP readings, protocol driven advice to GP to intensify treatment each month if targets not met<br>Depression: Regular monitoring of PHQ9 score and review and intensification of treatment if no improvement
Evidence-based guidelines and protocols	Healthlines advisors' scripts all based on careful review of national guidelines.<br>Encourage compliance with guidelines by sending GPs a simple flow chart summary with each treatment recommendation
Regular review	Healthlines advisors telephone patients monthly, based on scripts which raise new topics each month and review progress against goals
Promote medication adherence	Monthly review of medication adherence, scripts use evidence based strategies to improve adherence, advice to GPs by email if patients are non-adherent
Share recommendations with patients	Patients are given online access to guidelines and treatment recommendations sent to GPs
**Care co-ordination**	
Multi-component	Intervention combines interactive patient web portal, self-monitoring,

For peer review only

interventions	and telephone support from health advisor
Shared records	At onset, Healthlines receives information about patients from primary care records. All treatment recommendations shared with both primary care provider and patient. CVD: A summary of recent BP records from patient web-portal is sent to GP when treatment change is recommended.
Communication between the telehealth provider and primary care	Ideally, Healthlines advisors would visit general practices to build relationships, facilitate engagement with telehealth, resolve problems, but this was not achieved in this trial.
Regular monitoring of system performance	Reporting module which allows monitoring of management program (e.g. of number of patients who have been telephoned, number actively self-monitoring BP, number participating in on line cognitive behaviour therapy)
Support rather than duplicate primary care	All communications with primary care providers and patients reiterate the message that Healthlines is designed to support GPs in their role of managing patients. All treatment recommendations are made to GPs and copied to patients.
**Partnership**	
	All communications are shared. Communication is two way: GPs can contact Healthlines e.g. to change a patient's BP target
	GPs and service managers involved in designing the Healthlines intervention
**Context**	
	The nature and intensity of the intervention is tailored to the nature and severity of the patient's health condition.
	Patients are only invited to participate if they are above a specified severity threshold
	Recognizing that in the NHS patients have an enduring relationship with their GP, which reinforces the importance of supporting rather than duplicating or undermining that role
	Not all patients have access to reliable internet connections, so this intervention is only likely to be relevant to a proportion of those in need. Provide technical support to help patients, for example, log in to web portal. In evaluation, it is important to describe the characteristics of patients who take part.

For peer review only

For peer review only

Appendix 2 Web-portal for patients in cardiovascular intervention

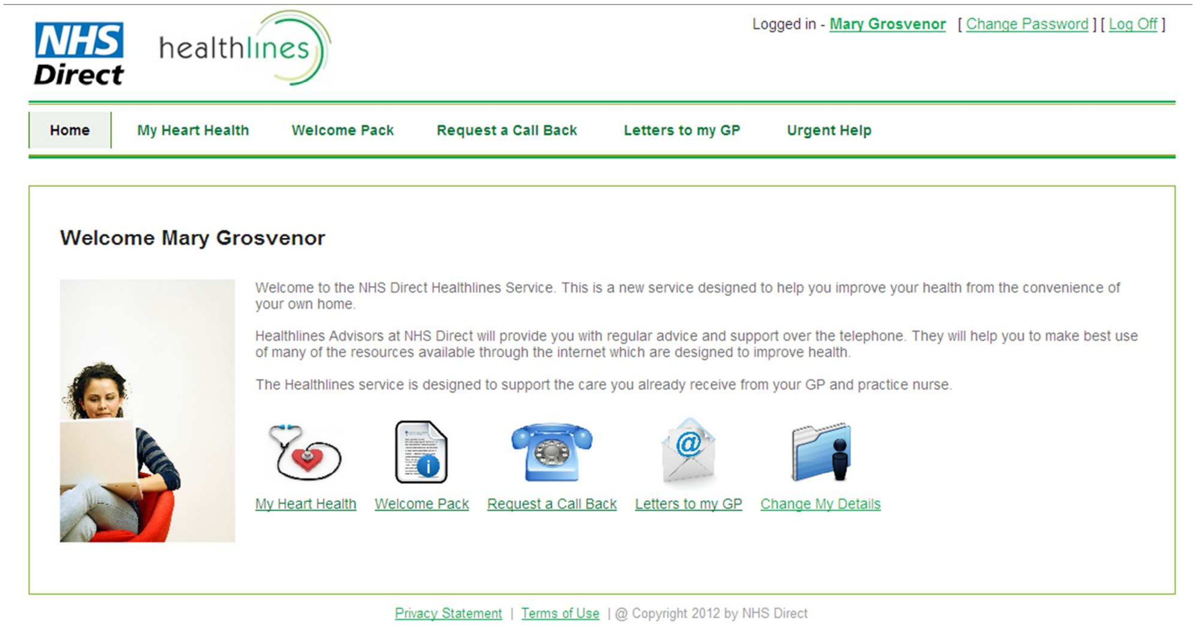

review only

Appendix 3 Blood pressure self-monitoring system

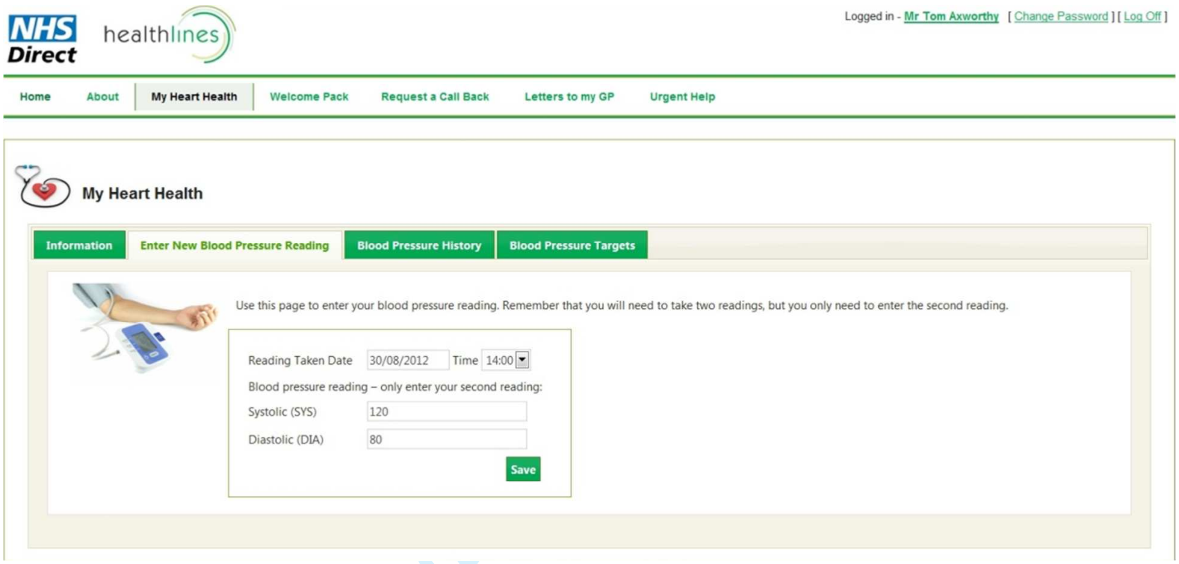

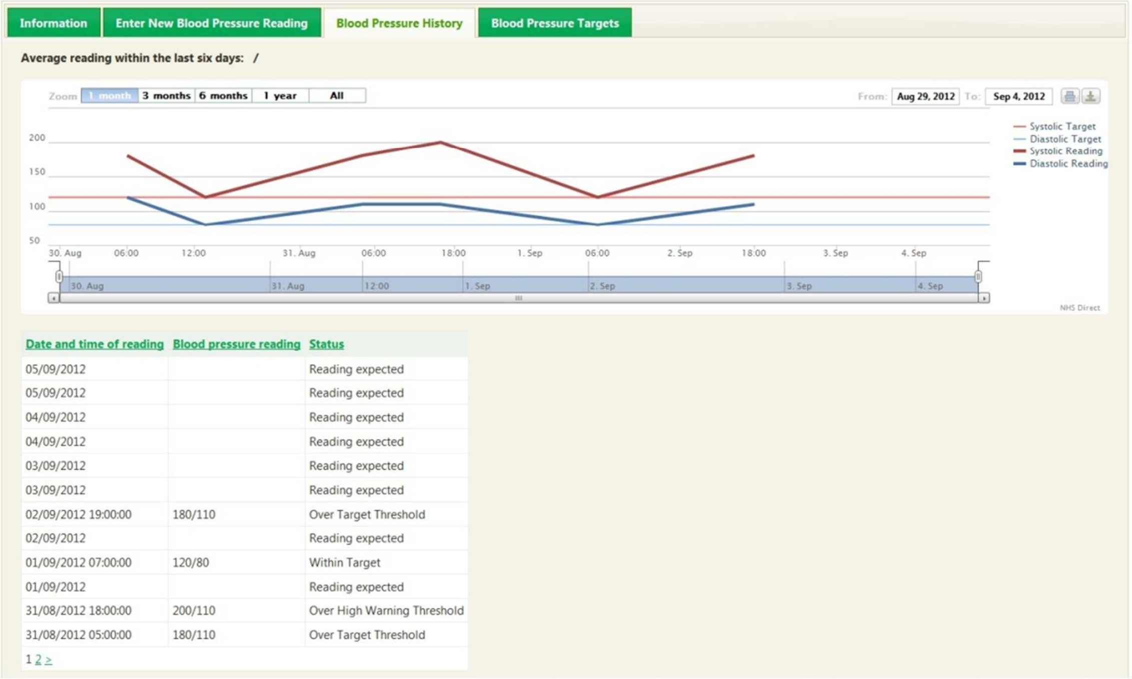

BMJ Open

TElehealth in CHronic disease: mixed-methods study to develop the TECH conceptual model for intervention design and evaluation

Journal:	*BMJ Open*
Manuscript ID:	bmjopen-2014-006448.R1
Article Type:	Research
Date Submitted by the Author:	10-Dec-2014
Complete List of Authors:	Salisbury, Chris; University of Bristol, Centre for Academic Primary Care<br>Thomas, Clare; University of Bristol, Centre for Academic Primary Care<br>O'Cathain, Alicia; The University of Sheffield, School of Health and Related Research (ScHARR)<br>Rogers, Anne; University of Southampton, Faculty of Health Sciences<br>Pope, Catherine; University of Southampton, Health Sciences<br>Yardley, Lucy; University of Southampton, Academic Unit of Psychology<br>Hollinghurst, Sandra; University of Bristol, Centre for Academic Primary Care<br>Fahey, Tom; Royal College of Surgeons in Ireland, HRB centre for Primary Care Research, Medical School, Department of General Practice<br>Lewis, Glyn; UCL Psychiatric Epidemiology, Mental Health Sciences Unit<br>Large, Shirley; NHS Direct,<br>Edwards, Louisa; University of Bristol, Centre for Academic Primary Care<br>Rowsell, Alison; University of Southampton, Health Sciences<br>Segar, Julia; University of Manchester, Centre for Primary Care<br>Brownsell, Simon; University of Sheffield, School of Health and Related Research (ScHARR)<br>Montgomery, Alan; University of Nottingham,
**Primary Subject Heading**:	General practice / Family practice
Secondary Subject Heading:	Health informatics, Health services research, Mental health, Cardiovascular medicine
Keywords:	Telemedicine < BIOTECHNOLOGY & BIOINFORMATICS, PRIMARY CARE, Hypertension < CARDIOLOGY, Organisation of health services < HEALTH SERVICES ADMINISTRATION & MANAGEMENT, Depression & mood disorders < PSYCHIATRY

SCHOLARONE™
Manuscripts

TElehealth in CHronic disease: mixed-methods study to develop the TECH conceptual model for intervention design and evaluation

Authors names, degrees, affiliations

Professor Chris Salisbury, MD FRCGP [1]
Dr Clare Thomas, PhD[1]
Professor Alicia O'Cathain, PhD [2]
Professor Anne Rogers, PhD [3]
Professor Catherine Pope, PhD [3]
Professor Lucy Yardley, PhD [4]
Dr Sandra Hollinghurst, PhD[1]
Professor Tom Fahey, MD [5]
Professor Glyn Lewis, PhD FRCPsych [6]
Dr Shirley Large, PhD [7]
Dr Louisa Edwards PhD [1]
Ms Alison Rowsell, MPhil [4]
Dr Julia Segar, PhD [8]
Dr Simon Brownsell, PhD [2]
Professor Alan A Montgomery, PhD [9]

[1] **University of Bristol**
Centre for Academic Primary Care, School of Social and Community Medicine
Canynge Hall, 39 Whatley Road, Bristol, BS8 2PS

[2] **University of Sheffield**
Medical Care Research Unit, School of Health and Related Research (ScHARR)
Regent Court, 30 Regent Street, Sheffield, S1 4DA

[3,4] **University of Southampton**
[3] School of Health Sciences, Building 67, Highfield, Southampton, Hampshire, S017 1BJ
[4] Centre for Applications of Health Psychology, Building 44, Highfield, Southampton SO17 1BJ

[5] **HRB Centre for Primary Care Research, Royal College of Surgeons in Ireland**
Medical School, Department of General Practice, 123 St Stephens Green, Dublin 2, Ireland

[6] **University College London**
Division of Psychiatry, University College London
67-73 Riding House Street, London, W1W 7EJ

[7] **NHS Direct**

For peer review only - http://bmjopen.bmj.com/site/about/guidelines.xhtml

Strawberry Fields, Berrywood Business Village, Tollbar Way,
Hedge End, Hampshire, SO30 2UN

[8] **The University of Manchester**
Centre for Primary Care, Institute of Population Health
5th Floor Williamson Building, Oxford Road, Manchester, M13 9PL

[9] **University of Nottingham**
Nottingham Health Science Partners, Nottingham Clinical Trials Unit
C Floor, South Block, Queen's Medical Centre, Nottingham, NG7 2UH

Corresponding author

Professor Chris Salisbury

Centre for Academic Primary Care, School of Social and Community Medicine, University of Bristol, Canynge Hall, 39 Whatley Road, Bristol, BS8 2PS, UK
Tel: +44 (0)117 331 4530 **Email:** c.salisbury@bristol.ac.uk

Keywords

Telemedicine, Chronic disease, Theoretical models, Cardiovascular diseases, Depression.

Words: 4858

For peer review only

Abstract

Objective: To develop a conceptual model for effective use of telehealth in the management of chronic health conditions, and to use this to develop and evaluate an intervention for people with two exemplar conditions: raised cardiovascular disease risk and depression.

Design: The model was based on several strands of evidence: a meta-review and realist synthesis of quantitative and qualitative evidence on telehealth for chronic conditions; a qualitative study of patients' and health professionals' experience of telehealth; a quantitative survey of patients' interest in using telehealth; and review of existing models of chronic condition management and evidence-based treatment guidelines. Based on these evidence strands, a model was developed and then refined at a stakeholder workshop. Then a telehealth intervention ('Healthlines') was designed by incorporating strategies to address each of the model components. The model also provided a framework for evaluation of this intervention within parallel randomized controlled trials in the two exemplar conditions and accompanying process and economic evaluations.

Setting: Primary care

Results: The TElehealth in CHronic Disease (TECH) model proposes that attention to four components will offer interventions the best chance of success: (i) engagement of patients and health professionals (ii) effective chronic disease management (including sub-components of self-management, optimization of treatment, care co-ordination) (iii) partnership between providers and (iv) patient, social and health system context. Key intended outcomes are improved health, access to care, patient experience and cost-effective care.

Conclusions: A conceptual model has been developed based on multiple sources of evidence which articulates how telehealth may best provide benefits for patients with chronic health conditions. It can be used to structure the design and evaluation of telehealth programs which aim to be acceptable to patients and providers, and cost-effective.

For peer review only - http://bmjopen.bmj.com/site/about/guidelines.xhtml

Article summary

Strengths and limitations of this study

- This paper describes the development and use of an evidence based conceptual model for the effective use of telehealth amongst patients with chronic conditions
- Having a conceptual model provides a framework for intervention development and evaluation
- The model is now being evaluated through parallel randomised controlled trials in two exemplar chronic conditions
- In order to develop a model which is clear, simple and generalisable there is a risk of over-simplification of the multiple mechanisms by which telehealth may have its effects
- The strength of evidence available to justify different components of the conceptual model is variable

For peer review only

For peer review only - http://bmjopen.bmj.com/site/about/guidelines.xhtml

Introduction

The role of telehealth in chronic health conditions

There is international interest in the potential of telehealth to support the management of patients with chronic health conditions. Telehealth refers to the use of electronic and telecommunication technologies to support health care at a distance from the patient. This reflects a recognition that, as the population ages, the needs of the increasing number of people with chronic conditions are likely to overwhelm the capacity of conventional health care services designed around scheduled one-to-one, face-to-face, appointments between patients and doctors. In the United Kingdom (UK) 30% of the population have at least one chronic condition and they account for 70% of total health services expenditure.[1] There is a need to harness the potential of technology to support people to manage themselves in their own homes. This has potential to shift the locus of control so that, through better access to information, people can become experts in their own care. Provision of health care at a distance (for example through telemonitoring) could in theory be more accessible, efficient and responsive than patients or professionals having to travel to face-to-face appointments.

Considerable resources have been committed to implementing different forms of telehealth for chronic conditions. For example, in the United States the Veterans Health Administration introduced a national home telehealth program which had enrolled about 50,000 patients by 2011,[2 3] the Renewing Health Consortium is developing and testing telehealth programme in nine European countries,[4] while in the UK the Whole System Demonstrator project was established to provide telehealth at scale for patients with conditions such as heart failure or chronic lung disease.[5-7]

Evidence of benefits

Although the potential benefits of telehealth in chronic condition management have been rehearsed for at least 20 years, evidence to support these arguments is limited.[8-10] Systematic reviews have been conducted in specific chronic conditions, along with overviews which have combined findings from a range of conditions, which have concluded that the evidence in favour of telehealth is weak and inconsistent. [8 9 11-16] Evidence of effectiveness is stronger for some conditions (for example, heart failure) than it is for others (for example, diabetes). Some studies report positive findings while others do not, and it has been difficult to identify a pattern in terms of disease, type of technology, or patient characteristics to explain these inconsistencies. There is a lack of evidence about mechanisms of action and about wider impacts of telehealth on utilization of other health care services.[9] There is inconsistent reporting of outcomes, suggesting a lack of clarity about the intended benefits of telehealth and making it difficult to compare studies. Evidence about cost-effectiveness or of successful wide scale implementation is particularly limited.

For peer review only - http://bmjopen.bmj.com/site/about/guidelines.xhtml

The need for a conceptual model

Telehealth is a complex intervention, [10 17] involving a number of interacting components, such as the type of technology, the infrastructure, the human support available, and the capabilities of the patient in relation to the technology. For any individual, telehealth is likely to be only one facet of the health care they receive, so telehealth cannot be understood in isolation from the health care system in which it is provided.

Over the last 15 years there has been increasing awareness of the importance of theory both in the development and evaluation of complex interventions.[18] Theory is needed in order to understand the relationship between context, mechanism of action and intended outcomes, but this has largely been neglected in the field of telehealth.[19-21] While there are well recognised theories in related topics such as behaviour change (e.g. the Theory of Planned Behavior[22], the Behaviour Change Wheel[23], Ritterbrand[24]), and why technologies get used (for example, the Technology Acceptance Model[25]), there is no over-arching theory which connects these and other elements (such as co-ordination between service providers) essential to chronic disease management in the context of telehealth.

What is needed is a clear conceptual model for how and why a telehealth intervention for patients with chronic conditions is intended to have specified beneficial effects. Making explicit the theoretical chain of causation by which an intervention is intended to lead to its effects focuses attention on the most important features of the intervention that need to be delivered for it to be effective. A conceptual model also provides a framework for evaluation by identifying the contextual factors, steps in the causal chain and most important outcomes that need to be assessed. To be practically useful, a conceptual model should be sufficiently generalizable to apply to a range of conditions, types of interventions and health care settings.

This paper describes the development of a conceptual model for the role of telehealth in the management of chronic conditions. This was developed to inform the design of an intervention to support people with two exemplar conditions: raised cardiovascular disease risk (due to risk factors such as hypertension, smoking, obesity and hyperlipidaemia) or depression. These exemplars were chosen to represent very different types of condition, which would test the generalizability of the model, but which are both common and where there was existing evidence that some forms of telehealth could be effective.[26 27] By taking into account the views of patients and providers and considerations about cost as well as evidence of effectiveness, the intention was to develop a model for interventions which are likely to be suitable for implementation at wide scale, acceptable to stakeholders, and cost-effective.

For peer review only - http://bmjopen.bmj.com/site/about/guidelines.xhtml

Methods

Evidence review

The model was based on several sources of evidence. The methods and results for each strand of evidence are summarized below, but are described in more detail elsewhere.

1) A **meta-review and realist synthesis** of existing quantitative and qualitative evidence on telehealth for chronic conditions.[16] This consisted of an overview of existing systematic reviews of telehealth interventions. We focused on reviews of chronic conditions generally rather than in relation to specific conditions. We included telephone and internet-based interventions (such as telecoaching, telephone/internet counselling and follow-up) and telemonitoring of symptoms and vital signs, but not telemedicine approaches where technologies are used to share information between healthcare providers. We searched Medline, CINAHL, Embase, AMED, PsycINFO, and the Cochrane Library databases for high quality systematic reviews about telehealth and chronic conditions published in English between January 2005 and March 2010. Two reviewers independently reviewed abstracts and (where relevant) full papers and extracted data onto a standardized form. We supplemented the meta-review with a new systematic review to look in more detail at studies of telehealth interventions focused on telehealth interventions for prevention of cardiovascular disease.[28] In addition we identified and reviewed published qualitative studies of patients' experience of using telehealth interventions. In total, we included 16 systematic reviews (representing 662 quantitative studies) and 29 qualitative studies. We combined these sources of data in a realist synthesis in which we sought to identify mechanisms of action for telehealth in chronic conditions. Realist synthesis is an approach to reviewing research evidence on complex interventions in order to provide an explanatory analysis for how and why they work (or don't work) in particular contexts or settings.[29]

2) A **qualitative study** of the potential role of telehealth in chronic conditions.[30] This involved interviews and observation with patients as well as doctors and nurses providing primary care for patients with chronic conditions, and health information advisors who provided an existing telephone based health coaching and care management service for patients with chronic conditions such as heart failure or diabetes.[31] Semi-structured interviews were conducted with 38 patients and 68 health professionals and observation was undertaken at a centre providing telehealth. The research took place between April 2010 and March 2011. Thematic analysis of qualitative data was undertaken.

3) A **survey of patients** to assess relationships between patient characteristics, health needs, difficulties with access to health care, attitudes towards and availability of various technologies, and interest in using different types of telehealth.[32] Patients

For peer review only - http://bmjopen.bmj.com/site/about/guidelines.xhtml

with either raised cardiovascular risk (n=872) or depression (n=606) were identified and randomly sampled from 34 general practices in two areas of the UK and invited to complete a questionnaire.

4) **Comparison with other models of chronic disease management**. In order to take account of and compare our emerging conceptual model with existing models and frameworks, we familiarised ourselves with other widely used models of chronic condition management, particularly (but not exclusively) those relating to the use of telehealth. We wanted to identify common factors in these models which appeared to be associated with improved care and benefits for patients.

5) **Analysis of national guidelines**. In order to apply the model to our exemplar conditions we identified the main recommendations and priorities for treatment from the current UK guidelines and compared these with guidelines from the US and Europe. We cross-referenced these recommendations with our meta-review to identify evidence for the effectiveness of telehealth interventions (for example, the use of online programs to deliver cognitive behavioural therapy for depression; the use of home monitoring of blood pressure in patients with hypertension).

Synthesis

We synthesized the findings from our evidence review in two stages. First, it was clear from the meta-review and the qualitative study that engagement from both patients and professionals appeared to be key to the success of a telehealth intervention. We therefore used a modified PRECEDE-PROCEED[33] approach to intervention development in which we used the insights from our evidence sources to map the predisposing, enabling and reinforcing factors that determine engagement with telehealth creating separate 'maps' for patients and health professionals. Predisposing factors provide the motivation to act in some way, enabling factors are those that make it possible to carry out the action, and reinforcing factors influence the likelihood that one will perform the behaviour in the future, based on positive or negative feedback. Through discussion within the research team, we listed and grouped themes from the literature reviews, qualitative research and patient survey, cross-referenced to the sources of evidence. Next, commonalities across these three sources of evidence were highlighted and key themes relating to engagement with telehealth were identified. These key themes were then independently organised into predisposing, enabling, and reinforcing factors by members of the research team familiar with the PRECEDE-PROCEED[33] definitions. Since it is possible that the same information can first serve as a predisposing factor, and then later as a reinforcing factor, differences in classification, although rare, were resolved through discussion. Nonetheless, the real importance of classifying information into these types of causal factors was to devise temporally-appropriate strategies to enhance motivators of and mitigate barriers to the target behaviour.

For peer review only - http://bmjopen.bmj.com/site/about/guidelines.xhtml

Second, we developed a draft model for the use of telehealth to support the management of chronic conditions which encapsulated the main findings from the evidence review. We discussed the findings from the various studies within the research team, seeking to describe hypothesised relationships between different constructs in a schematic manner. Several different layouts and versions of the model were discussed iteratively in meetings of the research team as we critiqued and sought to improve the model. Finally, we convened an intensive one day workshop for a wide range of stakeholders (n= 38) including patients, care providers, managers, commissioners of services, independent academics and the research team. We presented the findings of the evidence review and the draft model to the stakeholders, who discussed it in small groups and provided feedback. We used this to refine the final model, which we labelled the TECH model (**TE**lehealth for **CH**ronic Diseases).

Using the model to design an intervention

The research team used the TECH conceptual model to design a telehealth intervention known as the Healthlines Service. This was designed to be delivered by NHS Direct, which (at the time the intervention was designed) provided health information and advice throughout England based on a network of telephone call centres and an associated website. The intention was to design an intervention that would be likely to be cost-effective by maximizing patient benefit at minimum cost, and which could feasibly be rolled out quickly on a national scale if it proved to be effective. For these reasons, the design of the intervention sought to incorporate technologies which were already available and approaches for which there was already some evidence of effectiveness. We avoided cutting-edge technologies that were not already developed or tested and high cost solutions that would be unlikely to be widely available or deliverable to large numbers of patients. In order to maximize population benefit the aim was to focus on the large number of patients at moderate risk of health problems (e.g. patients with hypertension and other cardiovascular risk factors) rather than the small number of patients at high risk (for example, patients who have already had a stroke).

The research team used the patient and health professional 'maps' generated through the PRECEDE-PROCEED method to develop strategies to promote engagement with the telehealth intervention by addressing each of the predisposing, enabling and reinforcing factors previously identified.

The model as a framework for evaluation

The TECH conceptual model was used to provide a framework for evaluation by describing the extent to which each element of the model was successfully delivered and the intended outcomes were achieved. The Healthlines Service is being evaluated within two pragmatic parallel randomized controlled trials and accompanying process and economic evaluations. We recruited 43 general practices providing primary health care in three areas of England. Adult patients from these practices with either (a) raised risk of a first cardiovascular event (10 year risk >20%) or (b) depression were recruited to take part and were individually

For peer review only - http://bmjopen.bmj.com/site/about/guidelines.xhtml

randomized to receive either usual primary care plus extra support from the Healthlines Service or usual primary care alone. The protocol for these trials has been published (Trial Registration: Current Controlled Trials: cardiovascular disease risk trial ISRCTN27508731 and Depression trial ISRCTN14172341).[34]

Results

Evidence review

Meta-review, realist synthesis, qualitative study and quantitative patient survey:

Key findings from these studies are summarized in Box 1.

Existing models of chronic condition management

We identified a number of existing models for the management of chronic conditions, but the dominant approach is the Chronic Care Model (CCM).[35] A number of studies have suggested that programmes based on the CCM can improve health outcomes for a range of chronic conditions, although it is uncertain which components of the model are most important or whether all are necessary.[36-38] The CCM includes elements which relate to national aspects of the health care system and does not in itself provide a model for the design of telehealth interventions. Between 2003 and 2007, the Veterans Administration introduced a national home telehealth program, Care Coordination/Home Telehealth (CCHT),[2] which was strongly influenced by the CCM but applied the concepts more specifically to telehealth applications in a United States context.

Review of national guidelines

In order to apply a conceptual model to a specific condition the key health problems and care needs must be identified. For raised cardiovascular disease risk, international guidelines suggested that these were the modifiable risk factors of hypertension, smoking, obesity, raised cholesterol, and lack of exercise.[39-43] Evidence based priorities for intervention included optimizing drug treatment in order to achieve blood pressure targets, ensuring medication adherence, providing nicotine replacement therapy for smokers along with behavioural support, providing advice about diet and exercise and referral to weight management programs for obesity, and ensuring that statins were prescribed and taken.

For depression, the priorities for intervention included offering psychological therapies such as cognitive behavioural therapy and/or anti-depressant drug treatment with intensity of treatment tailored in relation to need, having relapse prevention strategies, ensuring medication adherence, offering peer support, avoiding alcohol misuse, encouraging exercise, and assessing suicidal risk.[44 45]

For peer review only - http://bmjopen.bmj.com/site/about/guidelines.xhtml

Box 1: Key findings from the meta-review, qualitative study and patient survey

Meta-review[16 28]

- Some evidence of improvements in clinical outcomes
- Much of the primary research is of poor quality and limited to short term effects
- Evidence about impact on the wider health care system and cost-effectiveness is sparse
- Inconsistent findings about effectiveness and resource utilization, with few clear patterns in terms of types of patient, disease or technology associated with benefits
- Many telehealth interventions for chronic conditions have struggled to engage both patients and health care professionals, with low uptake and high drop-out rates
- Simple technologies, especially those based on telephone support, have at least as strong an evidence base as more sophisticated technologies such as tele-monitoring
- Telephone support seems to enhance the benefit of web based technology

Realist synthesis

This suggested three key mechanisms by which telehealth worked to improve health outcomes:

- Relationships: good connections between patients, peer groups and/or professionals provide support
- Fit: Acceptability, ease of use, and integration into everyday routines were important to both patients and professionals
- Visibility: Monitoring provides feedback, reinforcement and prompts to change behaviour but can also have negative connotations of surveillance

Qualitative study[30]

- Nurses and doctors working in primary care were ambivalent about the contribution of telehealth to chronic condition management, because of concerns about the lack of evidence of benefit, duplication of their own work and a threat to their role
- There is a need to take account of how new telehealth programs integrate with existing health system structures
- Patients were more likely to trust a telehealth system if it is endorsed by their usual primary care providers
- Patients valued a personal approach based in human interaction

Patient survey[32]

- There was moderately strong interest in telehealth support for chronic conditions across all age groups
- There was greatest interest in telephone and internet based interventions, and minimal interest in social media, particularly amongst older patients with chronic conditions
- There was little relationship between health care need or difficulties in accessing health care and interest in telehealth
- The most important constructs associated with interest in telehealth were confidence in using the technology and perceived advantages and disadvantages of telehealth
- Interest in telehealth was not related to patient socio-demographic variables, after adjusting for modifiable factors such as access to and confidence in using the technology

For peer review only - http://bmjopen.bmj.com/site/about/guidelines.xhtml

Synthesis and developing the model

Figure 1 shows the final TECH model illustrating the key components, and relationships between them, which we hypothesise will deliver cost effective improvements in chronic disease management using telehealth. In summary, this model proposes that interventions to promote self-management, optimisation of treatment and care co-ordination are all essential aspects of chronic disease management, which are likely to lead to improved health outcomes, patient experience, access to care and more cost-effective delivery of care. These benefits are more likely to be achieved if the service is delivered in an integrated way with other health care providers, and the effectiveness of telehealth is likely to be moderated by the extent of patient and provider engagement and also moderated by characteristics of patients and the health care system.

These components are described in more detail below.

1. Engagement of patients and primary care providers

The literature meta-review highlighted that many telehealth interventions have been unsuccessful because of low uptake by patients and high rates of drop-out. Both our qualitative research and the patient survey illustrated the range of factors that act as motivators or barriers to patients using telehealth. These are summarized in Box 2 based on our PRECEDE-PROCEED map of predisposing, enabling and reinforcing factors for patients.

Box 2: Predisposing, enabling and reinforcing factors to the use of telehealth by patients

Pre-disposing

- Attraction of having support for health problems on demand, having more time, getting greater support
- Patients having a clear understanding of why they have been offered telehealth treatment
- Confidence in ability to use the technology
- Being reassured about privacy and confidentiality

Enabling

- Good access to fast reliable internet connection
- Technology which is simple and inexpensive, not complicated to use

Reinforcing

- Benefits of having regular review
- Importance of self-monitoring which promotes continued engagement
- Encouraging patient activation and involvement rather than passive reminders

For peer review only - http://bmjopen.bmj.com/site/about/guidelines.xhtml

With regard to health care professionals, our qualitative research indicated that many were unenthusiastic and, in some cases, resistant towards telehealth interventions. Our PRECEDE-PROCEED map for professionals identified several factors that were likely to influence engagement in telehealth. These included the belief that medicine should be evidence-based, and scepticism about the evidence for telehealth (pre-disposing factor), concerns about duplication of care (pre-disposing), the need for technology to be simple and reliable (enabling), and the importance of clarity of roles for conventional and telehealth providers and good communication between them (reinforcing).

2. Effective chronic disease management:

Our evidence synthesis and review of existing models of chronic condition management suggested that strategies that contribute to effective care and which could be delivered via telehealth can be summarized under three headings: promoting self-management, optimizing treatment, and care coordination. The various strategies that comprise each of these headings are shown in Box 3, along with citations for specific studies or reviews that provide evidence of effectiveness for each element (not necessarily in the field of telehealth).

For peer review only

For peer review only - http://bmjopen.bmj.com/site/about/guidelines.xhtml

Box 3: Components of effective chronic condition management

Promoting self-management

- Behaviour change techniques, e.g. stimulus control, problem solving, cognitive restructuring, goal setting[46 47]
- Self-monitoring [27 48 49]
- Provide patient information [50 51]
- Promote self-efficacy [52-54]
- Shared decision making [51]
- Motivational interviewing [46 47]
- Personal support from health professionals [55 56]

Treatment optimisation

- Risk stratification with case management for complex patients [39 57]
- Treatment intensification [39 44 56 58]
- Use of evidence-based guidelines and protocols [44 56]
- Regular review [39 51 58]
- Promote medication adherence [47 51]
- Share treatment recommendations with patients [59]

Care co-ordination

- Interventions that included multiple reinforcing components [47 51 55]
- Shared records, information and treatment recommendations between patients, primary care and the telehealth provider [2 54]
- Communication (remote and face-to-face) between the telehealth provider and primary care [2]
- Regular monitoring of system performance [38 60]
- Seek to support rather than duplicate primary care [61]

3. Partnership

Our qualitative research highlighted that a telehealth intervention is just one aspect of the health care provided to a patient with a chronic condition. These patients are likely to continue to get the majority of their care from their family practitioner, with whom they may have had a long-term relationship, and whom they will continue to consult for reasons apart from their chronic condition. In addition, many patients with chronic conditions are likely to be receiving help from hospital specialists and other health and social care agencies.

However, our evidence review suggested that many previous telehealth interventions appear to have failed because they were designed in isolation from the rest of the health care system, leading to duplication of effort, lack of co-ordination between providers, inefficiency and confusion for patients. This is likely to reinforce the resistance expressed by

For peer review only - http://bmjopen.bmj.com/site/about/guidelines.xhtml

other health care providers. Our qualitative research showed that these other providers may perceive the telehealth intervention to be an unnecessary interference in their area of responsibility, possibly representing a threat to their future role.

Therefore, it is important for a model for telehealth interventions to emphasize that telehealth should be delivered in partnership, identifying the role that telehealth can play to support rather than compete with patients' main primary health care providers.

4. Context: characteristics of patients and wider social and health system

The patient survey and the literature review both indicated that characteristics of patients are likely to have an impact on how telehealth affects outcomes. These include socio-demographic characteristics, particularly age, the nature of their chronic condition, and the severity of their condition. The design of a telehealth intervention must also take account of the wider social and health system context.[62 63] For example a program designed to work within a health system context with a strong primary care foundation may need different features from one designed for a system in which patients consult different hospital specialists for each of their chronic conditions. Similarly, a system which assumes that patients have access to fast and reliable internet connections will not work where this does not apply. Finally, different funding models for health care create different financial incentives for providers and patients which may have a major influence over how telehealth systems are implemented.

Specifying outcomes

The TECH model depicted in Figure 1 seeks to capture the four components of the model in a way that is conceptually clear, simple and generalizable. It also proposes the improved outcomes that telehealth interventions are intended to deliver for patients with chronic conditions. These are improved health outcomes, access to care and patient experience, care provided in a way which is cost-effective. One criticism of earlier research on telehealth interventions has been the lack of consistency in reporting outcomes,[8] and this model provides a framework for the outcomes that should be assessed in future evaluations, as well as potential mediators in order to gain understanding of the mechanism of action.

Using the model to develop a telehealth intervention

We used the conceptual model to develop telehealth intervention programs to support the management of patients with (a) raised cardiovascular risk or (b) depression. We used the same model to design interventions which were similar in concept but different in terms of detailed content to address each of the priority health and care needs for these two groups of patients, based on our review of national guidelines.

Table 1 provides examples of how we devised strategies to be delivered within the Healthlines Service to populate the conceptual model for the intervention to be used for cardiovascular risk. Appendix 1 provides an expanded and more comprehensive list of the

For peer review only - http://bmjopen.bmj.com/site/about/guidelines.xhtml

strategies we used for both raised cardiovascular risk and depression, and the Healthlines Service has also been described in detail elsewhere.[34]

Table 1 Use of the TECH model to design the Healthlines telehealth intervention for patients with raised cardiovascular risk

MODEL ELEMENT	STRATEGIES INCLUDED IN INTERVENTION
**Engagement**	
Patient	Healthlines advisors provide simple welcome pack and technical support to overcome lack of confidence in technology
	Encourage sense of personal care through seeking to maximize continuity of care from one named Healthlines advisor
Health professional	All communications seek to reinforce message that the Healthlines Service is supporting and delivered alongside primary care
	Messages to primary care emphasize evidence based nature of interventions and guidance
**Promoting self-management**	
Behaviour change techniques	Healthlines cardiovascular intervention adapted from the Duke self-management package,[64] which uses scripts for advisors based on psychological principles of behaviour change. Intervention is tailored to patient's needs and goals.
Self-monitoring & feedback	Provide patients with free BP monitors and web-site to log readings which gives immediate feedback and graphical display about whether BP is above or below target (Appendices 2 and 3)
Provide patient information	Healthlines advisor works with patients to identify goals and then emails them links to further resources available on the internet, which have been quality assessed (e.g. diet advice, risk calculators, videos, patient forums)
**Treatment optimization**	
Risk stratification	Calculate cardiovascular risk. Level of intervention guided by level of risk factor with escalation to GP for patients at high risk
Treatment intensification	Monthly review of BP using online log of BP readings, protocol driven advice to GP to intensify treatment each month if targets not met
Promote medication adherence	Monthly review of medication adherence, scripts use evidence based strategies to improve adherence, GPs advised by email if patients appeared to be non-adherent
**Care co-ordination**	
Shared records	All treatment recommendations shared with both primary care provider and patient. A summary of recent BP records from patient web-portal is sent to GP when treatment change is recommended.
Regular monitoring of system performance	Reporting module which allows monitoring of management program (e.g. of number of patients who have been telephoned, number actively self-monitoring BP)
**Partnership**	
	All communications are shared between Healthlines, GP and patient. Communication is two way: GPs can contact Healthlines e.g. to change a patient's BP target
	GPs and service managers involved in designing the Healthlines intervention
**Context**	
	Not all patients in UK have access to reliable internet connections. It is important to describe the characteristics of patients who take part, for evaluation.

For peer review only - http://bmjopen.bmj.com/site/about/guidelines.xhtml

Use of the TECH model for evaluation

The TECH model proposes four main outcomes resulting from telehealth interventions for chronic disease, the first of which is improved health outcomes. For the cardiovascular trial the primary outcome is cardiovascular risk status 12 months following randomization. For depression, the primary outcome is a clinically significant improvement in depression. Secondary outcomes for both trials include health related quality of life, measures of access to health care and patient satisfaction with care. An economic analysis will assess cost-effectiveness over the 12 months of the trial, and for the cardiovascular risk trial will also model the long-term costs and benefits of the intervention after taking into account the predicted number of strokes and heart attacks over the next 10 years.[34]

Alongside the randomized controlled trial, a process evaluation will explore the extent to which the intervention was delivered as intended, and whether it led to the expected changes at each step of causal chain hypothesized by the conceptual model. It assesses patient characteristics and health service context, patient and primary care engagement, patient self-management, treatment optimisation, care co-ordination and partnership with other health care providers, as well as the primary and secondary outcomes described above. These are assessed using validated measures where possible. Qualitative research through interviews with patients, primary care health professionals and Healthlines advisors are conducted to understand in greater detail how the service was delivered, barriers and facilitators to implementation, and how and why the intervention did or did not appear to be effective from the perspectives of those delivering and receiving it.

Discussion

Principal findings

This article describes the development of the TECH conceptual model for the effective use of telehealth amongst patients with chronic conditions and illustrates how it has been used to develop telehealth interventions for patients at either raised risk of cardiovascular disease or depression and design the evaluation of those interventions. If these evaluations in different chronic conditions are positive, this will provide support for the model about how this type of telehealth intervention works, suggesting it can then be applied to other chronic conditions.

Alternatively, if the intervention is unsuccessful, it will be possible to assess each of the processes in the hypothesised causal chain in order to determine whether the intervention was not delivered as intended, or whether the assumed causal relationships were incorrect. For example, the model posits that one way in which telehealth works is by allowing people to monitor their own health, which will lead to changes in their behaviour, which will have a positive impact on their health. Having a model highlights the need to assess the extent to which participants actually did self-monitoring as intended, whether this was associated

For peer review only - http://bmjopen.bmj.com/site/about/guidelines.xhtml

with behaviour change, and whether this led to improved health outcomes. This kind of approach provides a framework for correction and adaptation of an intervention through understanding which intervention components are more or less effective at impacting proximal outcomes in the causal chain.[65]

Strengths and limitations

The strength of this research is that we have used diverse sources of evidence to develop a conceptual model which creates a framework for intervention development and evaluation. Each of the components of the model can be justified from our own research and evidence from previous literature.

Although it is arguable that the TECH model could be applicable not only to telehealth but to all chronic disease management programmes, the model draws attention to topics which are particularly important for telehealth (such as the need for partnership with primary care providers and attention to patient engagement) but which have been neglected in many previous telehealth interventions.

Recognizing that the simplest models have the greatest utility, we sought to provide a simple graphical depiction of the hypothesised causal chain in a successful telehealth intervention. However, we recognize that the model diagram over-simplifies the multiple potential mechanisms by which a telehealth intervention may have its effect. There are likely to be associations and interactions between different elements of the model, and both recognized and unrecognized confounding factors. However, to indicate all of these potential relationships in the model would, in our view, reduce its usefulness in providing a framework.

A further limitation is that the strength of underlying evidence to support each of the components of the model is variable. For example, evidence of the benefit of patient self-monitoring is strong for some chronic conditions, but not all, and although providing patient information and shared decision making are viewed as important aspects of chronic condition management in the Chronic Care Model and other similar models, the evidence that these strategies lead to improved patient outcomes is limited. Nevertheless, we have sought to include components in the model where the overall weight of evidence supports their value.

Relationship to previous studies

There are several existing models of behaviour change based on psychological theory which have been applied to, or are relevant to, telehealth.[22-24] However, behaviour change is only one aspect of the TECH model, and this is not its main purpose. The TECH model is intended to provide a framework for the design and evaluation of telehealth services at scale within health care systems, taking into account a much wider range of factors such as the potential efficiencies gained through better co-ordination of services.

For peer review only - http://bmjopen.bmj.com/site/about/guidelines.xhtml

Several previous authors have argued for the importance of theory in designing telehealth interventions, from a range of perspectives,[19 60 66]and there are also existing frameworks for the assessment (rather than the design) of telehealth for chronic conditions, such as the Model for Assessment of Telemedicine (MAST).[67] The intervention which is most relevant to our study and well described in terms of its underlying theoretical basis is the Comprehensive Health Enhancement Support System (CHESS), an umbrella term for several e-health programs combining information, adherence strategies, decision-making tools and support services.[65 68] Like the Healthlines intervention described here, CHESS was developed by combining several intervention features each of which had some theoretical justification. However, CHESS was developed without any clear theory about how the program features related to each other,[65] and the TECH model underpinning the Healthlines intervention is intended to address this limitation. Greenhalgh et al have taken a more radical stance and argued against the quasi-experimental approach advocated by previous authors in favour of in-depth case studies, viewing program evaluation not as experimentation but as social practice.[69] They claim that there is a need to recognize the complex political dynamics and language games practiced by different stakeholders and to question rationalist assumptions about 'what works'.[69] We recognize the importance of these political considerations in how telehealth programs are implemented and evaluated, and in how the findings from such evaluations are sometimes interpreted to fulfil a prior agenda. However, this does not undermine the need to develop interventions based on a understanding of how and in what ways telehealth programs might be effective; indeed a clear theoretical basis for interventions and clarity about intended outcomes might provide the most robust defence against selective use of findings and may allow a more nuanced understanding about why interventions are more or less effective in different contexts.

Implications for clinicians and policy-makers

This paper describes a clear conceptual model, based on several sources of evidence, which helps to articulate the theoretical basis for how, why and under what circumstances telehealth could provide specified benefits for patients with chronic health conditions. Because it is based on evidence-based components and the views of stakeholders, the TECH model provides the basis for the design of telehealth interventions which are likely to be effective, cost-effective and acceptable to patients and health care providers. Importantly, it also provides a framework for evaluation of these interventions.

For peer review only - http://bmjopen.bmj.com/site/about/guidelines.xhtml

Funding

This article presents independent research funded by the National Institute for Health Research (NIHR) under its Programme Grant for Applied Research (grant reference RP-PG-0108-10011)

The views expressed in this publication are those of the author(s) and not necessarily those of the NHS, the NIHR, or the Department of Health.

Competing interests

The authors have no competing interests

Contributors

CS, A O'C, AR, CP, LY, SH, TF, GL, SL, SB and AM had the idea, developed the protocol, obtained funding and supervised the research. CT and LE managed the research programme. AR, CP and AO'C conducted the meta-review and evidence synthesis. AR and JS conducted the qualitative study. LE conducted the patient survey. All authors contributed to model development. CS drafted the paper which was critically reviewed by all authors. CS is guarantor.

Acknowledgements

We would like to thank the members of the Program Steering Committee, chaired by Professor Brian McKinstry, patient representatives, members of the program advisory group and other stakeholders who contributed to the different components of research which led to the model described in this paper. We would also like to thank Prof Hayden Bosworth for allowing us to adapt the Duke self-management system and NHS Direct for implementing the intervention which is the basis for the trial.

Data sharing

No additional data available.

Appendices

Appendix 1: Use of the model to design the Healthlines telehealth intervention for patients at raised cardiovascular risk or depression

Appendix 2 Web-portal for patients in cardiovascular intervention

Appendix 3 Blood pressure self-monitoring system

For peer review only - http://bmjopen.bmj.com/site/about/guidelines.xhtml

References

1. DH/Long Term Conditions. Long Term Conditions Compendium of Information

2012.
https://www.gov.uk/government/uploads/system/uploads/attachment_data/file/216528/dh_134486.pdf

2. Darkins A, Ryan P, Kobb R, et al. Care Coordination/Home Telehealth: the systematic implementation of health informatics, home telehealth, and disease management to support the care of veteran patients with chronic conditions. Telemedicine and e-Health 2008:1118-26

3. Cruickshank J. Telehealth: what can the NHS learn from experience at the US Veterans Health Administration? 2012. http://www.2020health.org/dms/2020health/downloads/reports/Telehealth-VA/Telehealth%20VA.pdf.

4. Renewing Health: regions of Europa working together for health. http://www.renewinghealth.eu/en/home.

5. Cartwright M, Hirani SP, Rixon L, et al. Effect of telehealth on quality of life and psychological outcomes over 12 months (Whole Systems Demonstrator telehealth questionnaire study): nested study of patient reported outcomes in a pragmatic, cluster randomised controlled trial. BMJ 2013;**346**

6. Henderson C, Knapp M, Fernandez J-L, et al. Cost effectiveness of telehealth for patients with long term conditions (Whole Systems Demonstrator telehealth questionnaire study): nested economic evaluation in a pragmatic, cluster randomised controlled trial. BMJ 2013;**346**:f1035

7. Bower P, Kennedy A, Reeves D, et al. A cluster randomised controlled trial of the clinical and cost-effectiveness of a 'whole systems' model of self-management support for the management of long-term conditions in primary care: trial protocol. Implementation Science 2012;**7**(1):7

8. Wootton R. Twenty years of telemedicine in chronic disease management--an evidence synthesis. J. Telemed. Telecare 2012;**18**(4):211-20

9. Black AD, Car J, Pagliari C, et al. The impact of eHealth on the quality and safety of health care: a systematic overview. . PLoS Medicine / Public Library of Science 2011;**8**(1):e1000387

10. Car J, Huckvale K, Hermens H. Telehealth for long term conditions. BMJ 2012;**344** doi: 10.1136/bmj.e4201.

11. Murray E, Burns J, See TS, Lai R, Nazareth I. Interactive Health Communication Applications for people with chronic disease. Cochrane Database of Systematic Reviews 2005(4):CD004274

12. Barlow J, Singh D, Bayer S, Curry R. A systematic review of the benefits of home telecare for frail elderly people and those with long-term conditions. J Telemed.Telecare 2007;**13**(4):172-79

13. Pare G, Jaana M, Sicotte C. Systematic review of home telemonitoring for chronic diseases: the evidence base. J. Am. Med. Inform. Assoc. 2007;**14**(3):269-77

14. Deshpande A, Khoja S, McKibbon A, Jadad AR. Real-time (synchronous) telehealth in primary care: systematic review of systematic reviews. Technology Report No 100. Ottawa: Canadian Agency for Drugs and Technologies in Health, 2008.

For peer review only - http://bmjopen.bmj.com/site/about/guidelines.xhtml

15. Polisena J, Coyle D, Coyle K, McGill S. Home telehealth for chronic disease management: a systematic review and an analysis of economic evaluations. Int. J. Technol. Assess. Health Care 2009;**25**(3):339-49

16. Pope C, Rowsell A, O'Cathain A, Brownsell S. For want of evidence: a meta-review of home-based telehealth for the management of long-term conditions. 2011. http://www.bristol.ac.uk/healthlines/documents/popeetal.pdf.

17. Lilford RJ, Foster J, Pringle M. Evaluating eHealth: How to Make Evaluation More Methodologically Robust. PLoS Med 2009;**6**(11):e1000186

18. Campbell NC, Murray E, Darbyshire J, et al. Designing and evaluating complex interventions to improve health care. BMJ 2007;**334**(7591):455-59

19. Kaplan B. Evaluating informatics applications--some alternative approaches: theory, social interactionism, and call for methodological pluralism. Int J Med Inform 2001;**64**(1):39-56

20. Mackert M. Expanding the theoretical foundations of telemedicine. J. Telemed. Telecare 2006;**12**(1):49-50

21. Gammon D, Johannessen LK, Sorensen T, Wynn R, Whitten P. An overview and analysis of theories employed in telemedicine studies. A field in search of an identity. Methods Inf. Med. 2008;**47**(3):260-9

22. Ajzen I. The theory of planned behavior. Organ. Behav. Hum. Decis. Process. 1991;**50**(2):179-211

23. Michie S, van Stralen M, West R. The behaviour change wheel: A new method for characterising and designing behaviour change interventions. Implementation Science 2011;**6**(1):42

24. Ritterband LM, Thorndike FP, Cox DJ, Kovatchev BP, Gonder-Frederick LA. A Behavior Change Model for Internet Interventions. nn. Behav. Med. 2009;**38**(1):18-27

25. Davis FD. Perceived usefulness, perceived ease of use, and user acceptance of information technology. MIS Q. 1989;**13**(3):319-40.

26. Dellifraine JL, Dansky KH. Home-based telehealth: a review and meta-analysis. J Telemed.Telecare. 2008;**14**(2):62-66

27. Pare G, Moqadem K, Pineau G, St-Hilaire C. Clinical effects of home telemonitoring in the context of diabetes, asthma, heart failure and hypertension: a systematic review. Journal of medical Internet research 2010;**12**(2):e21

28. Merriel SWD, Andrews V, Salisbury C. Telehealth interventions for primary prevention of cardiovascular disease: A systematic review and meta-analysis. Prev. Med. 2014;**64C**(0)

29. Pawson R, Greenhalgh T, Harvey G, Walshe K. Realist synthesis: an introduction: ESRC Research Methods Programme, University of Manchester, 2004.

30. Segar J, Rogers A, Salisbury C, Thomas C. Roles and identities in transition: boundaries of work and inter-professional relationships at the interface between telehealth and primary care. Health & Social Care in the Community 2013;**21**(6):606-13

31. Steventon A, Tunkel S, Blunt I, Bardsley M. Effect of telephone health coaching (Birmingham OwnHealth) on hospital use and associated costs: cohort study with matched controls. BMJ 2013;**347:**f4585

For peer review only - http://bmjopen.bmj.com/site/about/guidelines.xhtml

For peer review only

32. Edwards L, Thomas C, Gregory A, et al. Are people with chronic diseases interested in using telehealth? J. Med. Internet Res. 2014; 16(5): e123

33. Green L, Kreuter M. *Health program planning: An educational and ecological approach.* . 4th ed. New York, NY: McGrawhill, 2005.

34. Thomas CL, Man MS, O'Cathain A, et al. Effectiveness and cost-effectiveness of a telehealth intervention to support the management of long-term conditions: study protocol for two linked randomized controlled trials. Trials 2014;**15**:36

35. Wagner EH, Austin BT, Von KM. Improving outcomes in chronic illness. Manag.Care Q. 1996;**4**(2):12-25

36. Singh D. Transforming Chronic Care: Evidence about improving care for people with long-term conditions: University of Birmingham, 2005.

37. Bodenheimer T. Interventions to improve chronic illness care: evaluating their effectiveness. Disease management : DM 2003;**6**(2):63-71

38. Ham C. The ten characteristics of the high-performing chronic care system. Health Economics, Policy and Law 2010;**5**(01):71-90

39. NICE. Hypertension: Clinical management of primary hypertension in adults. CG127 2011. www.nice.org.uk/guidance/CG127

40. NICE. Lipid Modification: cardiovascular risk assessment and the modification of blood lipids for the primary and secondary prevention of cardiovascular disease. Clinical Guideline 67. 2008.

41. Pearson TA, Blair SN, Daniels SR, et al. AHA Guidelines for Primary Prevention of Cardiovascular Disease and Stroke: 2002 Update: Consensus Panel Guide to Comprehensive Risk Reduction for Adult Patients Without Coronary or Other Atherosclerotic Vascular Diseases. Circulation 2002;**106**(3):388-91

42. Mosca L, Benjamin EJ, Berra K, et al. Effectiveness-Based Guidelines for the Prevention of Cardiovascular Disease in Women—2011 Update: A Guideline From the American Heart Association. Circulation 2011;**123**(11):1243-62

43. Graham I, Atar D, Borch-Johnsen K, et al. European guidelines on cardiovascular disease prevention in clinical practice: executive summary: Fourth Joint Task Force of the European Society of Cardiology and Other Societies on Cardiovascular Disease Prevention in Clinical Practice. Eur. Heart J. 2007;**28**(19):2375-414

44. NICE. Depression in adults. The treatment and management of depression in adults. Clinical guideline 90. 2009. http://www.nice.org.uk/CG90.

45. Work group on major depressive disorder. Practice guideline for the Treatment of Patients With Major Depressive Disorder. 2010. http://psychiatryonline.org/pb/assets/raw/sitewide/practice_guidelines/guidelines/mdd.pdf.

For peer review only

For peer review only - http://bmjopen.bmj.com/site/about/guidelines.xhtml

46. Greaves CJ, Sheppard KE, Abraham C, et al. Systematic review of reviews of intervention components associated with increased effectiveness in dietary and physical activity interventions. BMC.Public Health 2011;**11**:119

47. Katon WJ, Lin EH, Von KM, et al. Collaborative care for patients with depression and chronic illnesses. N.Engl.J.Med. 2010;**363**(27):2611-20

48. AbuDagga A, Resnick HE, Alwan M. Impact of blood pressure telemonitoring on hypertension outcomes: a literature review. [Review]. Telemedicine Journal & E-Health 2010;**16**(7):830-38

49. Agarwal R, Bills JE, Hecht TJ, et al. Role of home blood pressure monitoring in overcoming therapeutic inertia and improving hypertension control: a systematic review and meta-analysis. Hypertension 2011;**57**(1):29-38

50. Roumie CL, Elasy TA, Greevy R, et al. Improving blood pressure control through provider education, provider alerts, and patient education: a cluster randomized trial. Ann. Intern. Med. 2006;**145**(3):165-75

51. NICE. Medicines adherence: involving patients in decisions about prescribed medicines and supporting adherence. CG76. 2011. http://www.nice.org.uk/CG76.

52. Lorig KR, Ritter PL, Laurent DD, Plant K. Internet-based chronic disease self-management: a randomized trial. Med. Care 2006;**44**(11):964-71

53. Lorig KR, Holman H. Self-management education: history, definition, outcomes, and mechanisms. Ann Behav.Med 2003;**26**(1):1-7

54. Solomon M, Wagner SL, Goes J. Effects of a Web-based intervention for adults with chronic conditions on patient activation: online randomized controlled trial. Journal of medical Internet research 2012;**14**(1):e32

55. Bosworth HB, Olsen MK, Grubber JM, et al. Two Self-management Interventions to Improve Hypertension Control: a randomized trial. Ann. Intern. Med. 2009;**151**(10):687-95

56. Green BB, Cook AJ, Ralston JD, et al. Effectiveness of home blood pressure monitoring, Web communication, and pharmacist care on hypertension control: a randomized controlled trial.JAMA 2008;**299**(24):2857-67

57. Shea S, Weinstock RS, Starren J, et al. A randomized trial comparing telemedicine case management with usual care in older, ethnically diverse, medically underserved patients with diabetes mellitus. J. Am. Med. Inform. Assoc. 2006;**13**(1):40-51

58. Glynn LG, Murphy AW, Smith SM, Schroeder K, Fahey T. Interventions used to improve control of blood pressure in patients with hypertension. Cochrane Database of Systematic Reviews 2010(3):CD005182

For peer review only - http://bmjopen.bmj.com/site/about/guidelines.xhtml

59. McManus RJ, Mant J, Bray EP, et al. Telemonitoring and self-management in the control of hypertension (TASMINH2): a randomised controlled trial. Lancet 2010;**376**(9736):163-72

60. Catwell L, Sheikh A. Evaluating eHealth Interventions: The Need for Continuous Systemic Evaluation. PLoS Med 2009;**6**(8):e1000126

61. Bodenheimer T. The future of primary care: transforming practice. N. Engl. J. Med. 2008;**359**(20):2086, 89

62. McLean S, Protti D, Sheikh A. Telehealthcare for long term conditions. BMJ 2011;**342**

63. May CR, Finch TL, Cornford J, et al. Integrating telecare for chronic disease management in the community: what needs to be done? BMC health services research 2011;**11**:131

64. Bosworth HB, Olsen MK, Dudley T, et al. Patient education and provider decision support to control blood pressure in primary care: A cluster randomized trial. Am. Heart J. 2009;**157**(3):450-56

65. Pingree S, Hawkins R, Baker T, duBenske L, Roberts LJ, Gustafson DH. The Value of Theory for Enhancing and Understanding e-Health Interventions. Am. J. Prev. Med. 2010;**38**(1):103-09

66. Baker LC, Macaulay DS, Sorg RA, Diener MD, Johnson SJ, Birnbaum HG. Effects of Care Management and Telehealth: A Longitudinal Analysis Using Medicare Data. J. Am. Geriatr. Soc. 2013;**61**(9):1560-67

67. Kidholm K, Ekeland AG, Jensen LK, et al. A Model for Assessment of Telehealth Applications: MAST. Int. J. Technol. Assess. Health Care 2012;**28**(01):44-51

68. Gustafson D, Wise M, Bhattacharya A, et al. The effects of combining Web-based eHealth with telephone nurse case management for pediatric asthma control: a randomized controlled trial. Journal of medical Internet research 2012;**14**(4):e101

69. Greenhalgh T, Russell J, Ashcroft RE, Parsons W. Why national eHealth programs need dead philosophers: Wittgensteinian reflections on policymakers' reluctance to learn from history. Milbank Q. 2011;**89**(4):533-63

For peer review only

For peer review only - http://bmjopen.bmj.com/site/about/guidelines.xhtml

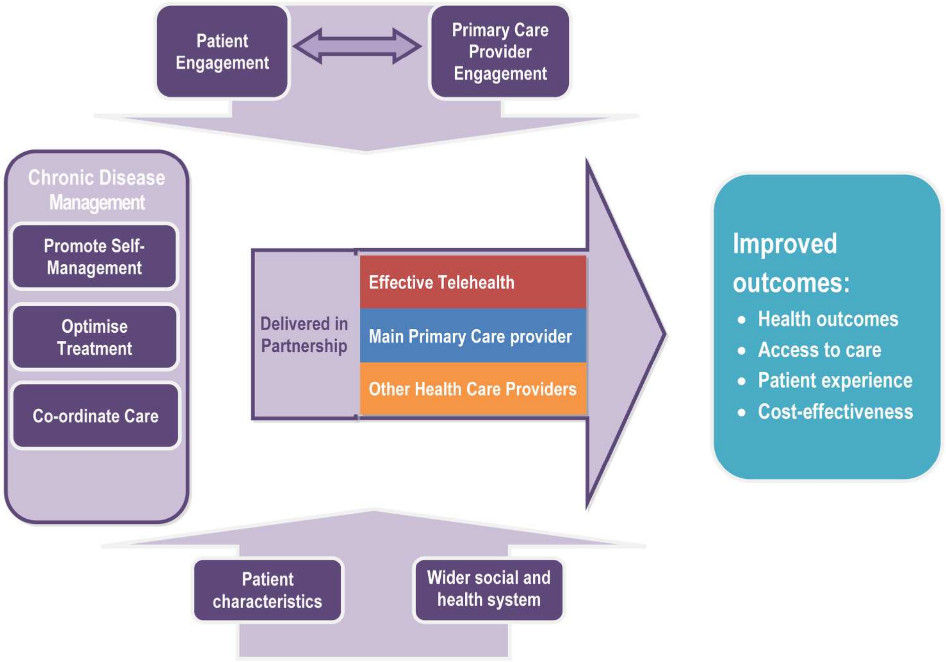

Figure 1. TECH Model for telehealth to support patients with chronic conditions
209x148mm (300 x 300 DPI)

view only

Appendix 1: Use of the model to design the Healthlines telehealth intervention for patients at raised cardiovascular risk or depression

MODEL ELEMENT	STRATEGIES INCLUDED IN INTERVENTION
**Engagement**	
Patient	Provide a 'Welcome Pack'. Emphasize that support with technology will be provided
	Healthlines advisors provide technical support e.g. with getting logged in to websites
	Promote the advantages to patients of using Healthlines, based on perceived advantages identified in qualitative research and other literature, and address perceived disadvantages
	Encourage sense of personal care through seeking to maximize continuity of care from named Healthlines advisor
	Regular positive reinforcement through monthly telephone calls from Healthlines advisor
	Encourage sense of partnership between patient, Healthlines and GP through frequent communication
Health professional	All communications seek to reinforce the message that the Healthlines service is supporting and delivered alongside primary care
	Regular communication with primary care
	Messages to primary care continually emphasize evidence based nature of interventions and guidance
**Promoting self-management**	
Behaviour change techniques	Healthlines cardiovascular intervention adapted from the Duke self-management package, which uses scripts for advisors based on psychological principles of behaviour change. Depression intervention encounters support use of the Living Life to the Full cognitive behaviour course, with additional modules relating to alcohol, exercise, relapse prevention. In both cases, intervention is tailored to patient's needs and goals.
Self-monitoring	CVD risk: Provide patients with free BP monitors and web-site to log readings<br>Depression: Patients using online Living Life to the Full regularly monitor their progress with self-assessment modules including score on PHQ9 questionnaire.
Feedback	CVD risk: BP website gives immediate feedback and graphical display about whether BP is above or below target and next actions
Provide patient	Healthlines advisor works with patients to identify goals and then emails

For peer review only

information	them links to further resources available on the internet which have been quality assessed (e.g. diet advice, risk calculators, videos, patient forums)
Promote self-efficacy	Using motivational interviewing approach, identify motivating factors, encourage action plans and goal setting
Motivational interviewing	All Healthlines advisors undertake motivational interviewing training
Shared decision making	Make targets (e.g. for BP) explicit to patients, provide information about advantages and disadvantages of treatments, encourage patients to discuss options with GP, share letters to GPs with patients
Personal support from health professionals	As far as possible, provide continuity of care from one named Healthlines advisor rather than an anonymous 'call-centre' approach
Peer support	Patients in depression intervention are offered option to access Big White Wall, an online forum for patients with depression
**Treatment optimization**	
Risk stratification	CVD: Calculate cardiovascular risk using QRISK. Level of intervention guided by level of risk factor with escalation to GP for patients at high risk<br><br>Depression: Assessment using PHQ9 and advice about treatment in relation to severity. PHQ9 also used to assess suicidal risk with use of a protocol for escalation and more detailed risk assessment for patients at significant risk
Treatment intensification	CVD: Monthly review of BP using online log of BP readings, protocol driven advice to GP to intensify treatment each month if targets not met<br><br>Depression: Regular monitoring of PHQ9 score and review and intensification of treatment if no improvement
Evidence-based guidelines and protocols	Healthlines advisors' scripts all based on careful review of national guidelines.<br><br>Encourage compliance with guidelines by sending GPs a simple flow chart summary with each treatment recommendation
Regular review	Healthlines advisors telephone patients monthly, based on scripts which raise new topics each month and review progress against goals
Promote medication adherence	Monthly review of medication adherence, scripts use evidence based strategies to improve adherence, advice to GPs by email if patients are non-adherent
Share recommendations with patients	Patients are given online access to guidelines and treatment recommendations sent to GPs
**Care co-ordination**	
Multi-component	Intervention combines interactive patient web portal, self-monitoring,

For peer review only

interventions	and telephone support from health advisor
Shared records	At onset, Healthlines receives information about patients from primary care records. All treatment recommendations shared with both primary care provider and patient. CVD: A summary of recent BP records from patient web-portal is sent to GP when treatment change is recommended.
Communication between the telehealth provider and primary care	Ideally, Healthlines advisors would visit general practices to build relationships, facilitate engagement with telehealth, resolve problems, but this was not achieved in this trial.
Regular monitoring of system performance	Reporting module which allows monitoring of management program (e.g. of number of patients who have been telephoned, number actively self-monitoring BP, number participating in on line cognitive behaviour therapy)
Support rather than duplicate primary care	All communications with primary care providers and patients reiterate the message that Healthlines is designed to support GPs in their role of managing patients. All treatment recommendations are made to GPs and copied to patients.
**Partnership**	
	All communications are shared. Communication is two way: GPs can contact Healthlines e.g. to change a patient's BP target
	GPs and service managers involved in designing the Healthlines intervention
**Context**	
	The nature and intensity of the intervention is tailored to the nature and severity of the patient's health condition.
	Patients are only invited to participate if they are above a specified severity threshold
	Recognizing that in the NHS patients have an enduring relationship with their GP, which reinforces the importance of supporting rather than duplicating or undermining that role
	Not all patients have access to reliable internet connections, so this intervention is only likely to be relevant to a proportion of those in need. Provide technical support to help patients, for example, log in to web portal. In evaluation, it is important to describe the characteristics of patients who take part.

For peer review only

Appendix 2 Web-portal for patients in cardiovascular intervention

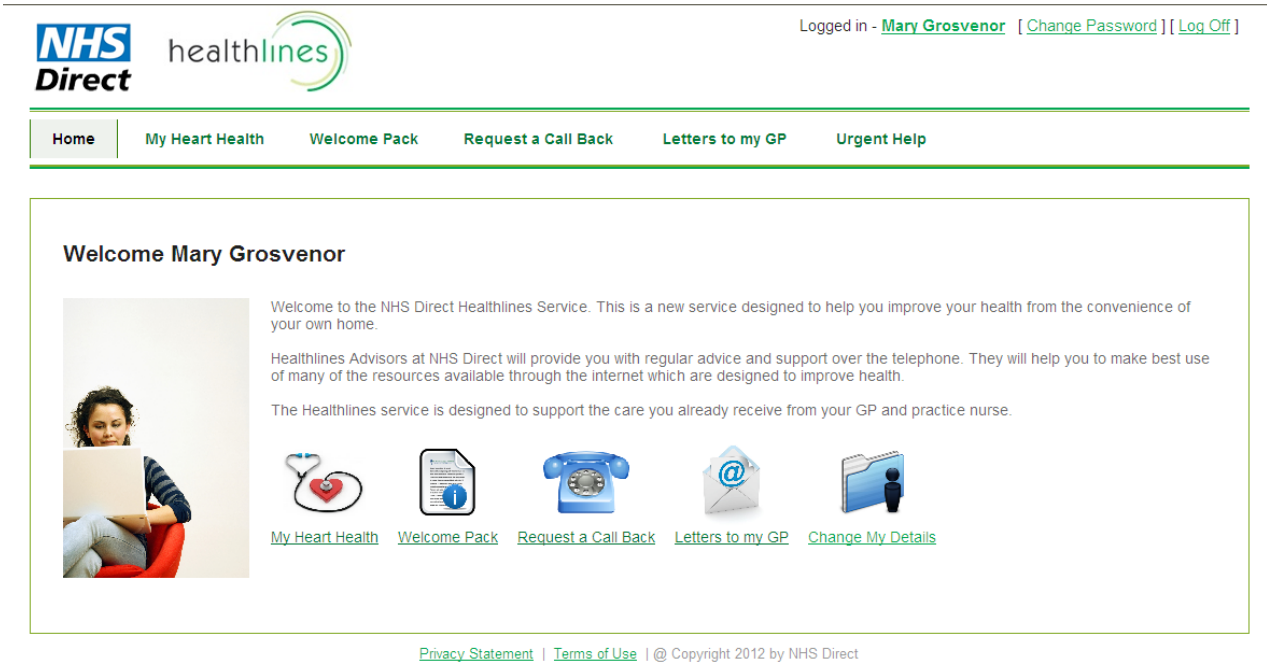

review only

Appendix 3 Blood pressure self-monitoring system

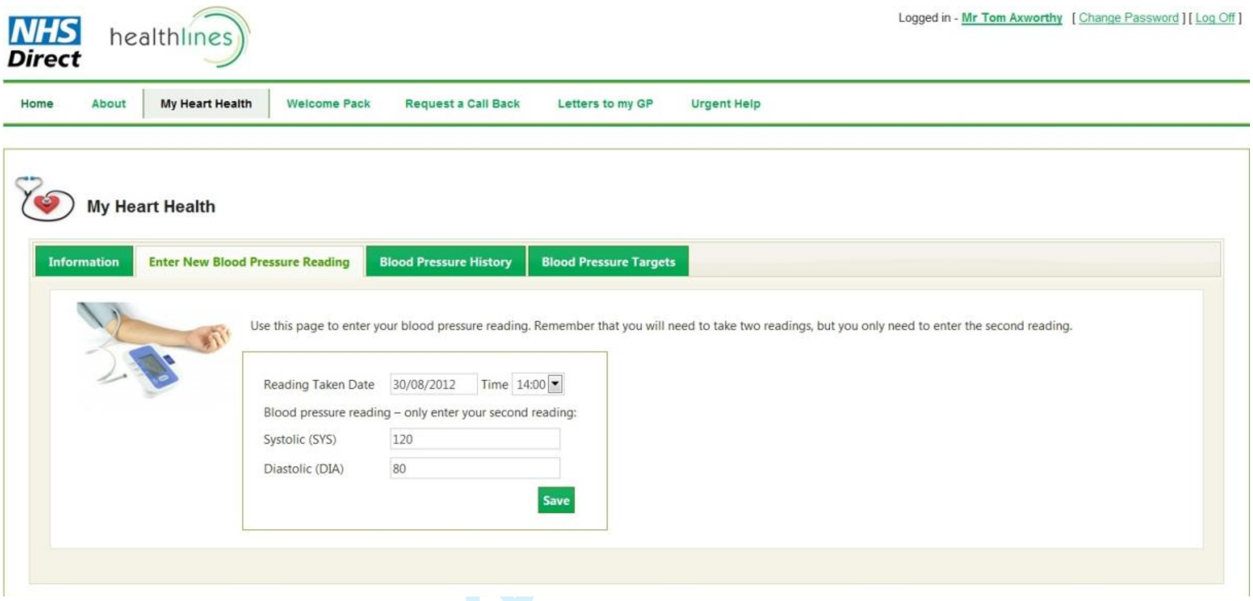

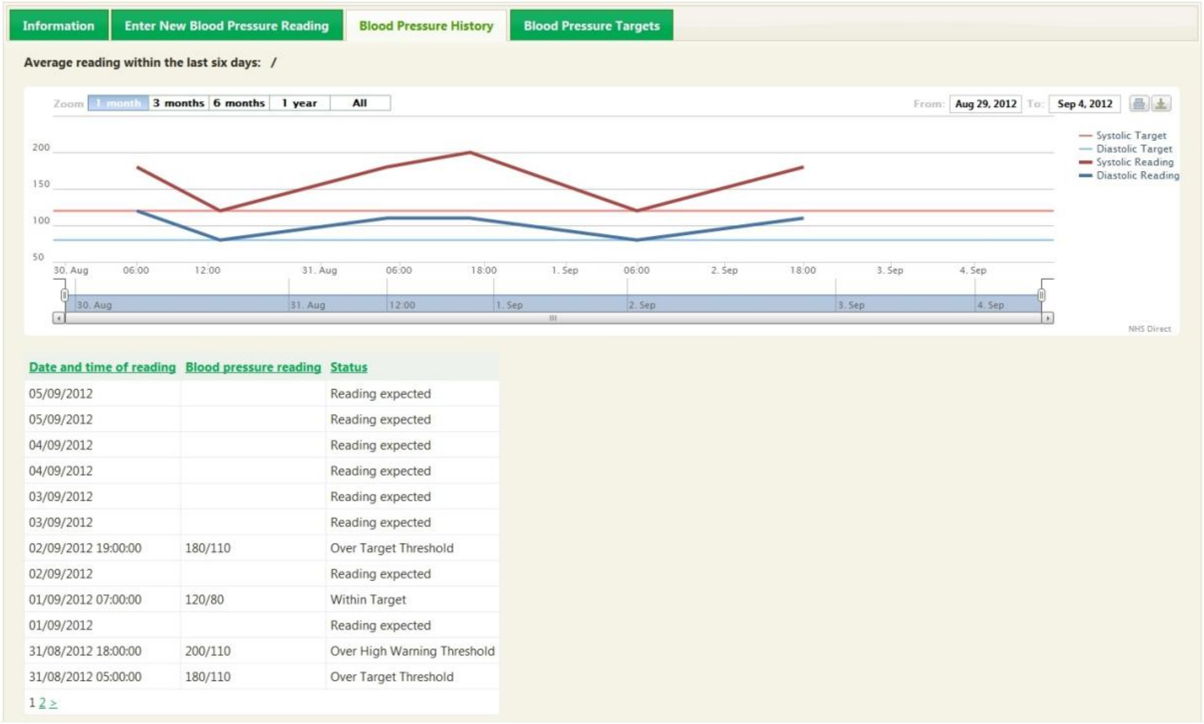

Supplement: Author's manuscript [file bmjopen-2014-006448.draft_revisions.pdf]
